# Characterization of the post-mating responses of *Drosophila hydei*, a species that lacks Sex-Peptide

**DOI:** 10.1038/s42003-026-10021-5

**Published:** 2026-04-11

**Authors:** Maxime Revel, Zeynep Yildirim, Léa Fabbro, Emi Nagoshi, Robert K. Maeda

**Affiliations:** Department of Genetics and Evolution, University of Geneva, Geneva, Switzerland

**Keywords:** Evolutionary biology, Behavioural ecology

## Abstract

Sex Peptide (SP) induces many of the most studied female post-mating responses (PMRs) in *Drosophila melanogaster* but has been lost multiple times in the *Drosophila* genus. We decided to explore the PMRs of *Drosophila hydei*, a species without *SP*. Our work shows that the PMRs in *D. hydei* are somewhat different than those found in *D. melanogaster* and may be the consequence of a selection for producing a reduced number of extremely long sperm. *D. hydei* females lack the substantial post-mating increase in egg production found in *D. melanogaster*, mostly displaying only a brief induction in the laying of stored eggs. Mated females do not show a reduction in lifespan that has been linked to changes in metabolism and egg production. To further explore the reproductive biology of this species, we performed sperm competition experiments that suggest that *D. hydei* females may select sperm based on characteristics linked to changes in seminal fluid proteins. This was further investigated by examining the structure of the seminal fluid-producing accessory glands and the egg laying PMRs in different *Drosophila* species. Finally, video-based monitoring of *D. hydei* females was used to uncover novel changes in circadian rhythm and light preference in mated females.

## Abstract (web summary)

Investigations on the post-mating responses of *Drosophila hydei* show significant differences from the model species *Drosophila melanogaster* and suggest that the selection for long sperm may be the primary force impacting male reproductive strategy.

## Introduction

Species each evolve reproductive strategies to effectively propagate their genome. Female post-mating responses (PMRs), where mating triggers changes in female physiology and behavior, are part of these strategies. The inducers of these PMRs are often found to be male seminal fluid products that are transferred to females during mating and have been identified in many species^1,2^. However, nowhere have PMRs been more studied than in the fruit fly *Drosophila melanogaster*, where it has been shown that the seminal fluid proteins (SFPs), produced by the male accessory gland (mAG), trigger a vast number of PMRs^2–4^.

Among the many *D. melanogaster* SFPs, the protein Sex Peptide (SP) has been found to induce a variety of PMRs, including: increasing egg production and laying, changing female receptivity to additional matings^5,6^, shifting the female circadian activity^7,8^, and shortening female lifespan^9,10^. Along with SP, other SFPs have been also found to be capable of inducing specific PMRs, for example, the Ovulin protein has been shown to be able to trigger the release of mature oocytes from the ovaries^11^, and the Acp36DE has been shown to be able to induce changes in the configuration of the uterus of the female^12^.

The large number of changes promoted by SP makes it one of the most potent PMR initiators. Accordingly, the *SP* gene is quite well conserved within the Drosophilids, unlike other PMR inducers like *ovulin* and *acp36DE,* which seem to be rapidly diverging and seem to be missing or poorly conserved outside of the melanogaster species group^13^. Yet despite its fundamental role in the reproductive biology of *D. melanogaster*, the *SP* gene has been lost in several branches of the *Drosophila* phylogenetic tree^14,15^, and there are indications that its role in species outside of *Sophophora* may be less prominent^16^. This begs the question of whether or not PMRs outside of *D. melanogaster* will be similar, and if so, how these PMRs would be induced.

At first glance, one might expect that certain PMRs would be found almost universally, even in the absence of SP. This is particularly true of PMRs that might economize the use of resources. For example, the induction of egg production and laying in mated females would seem to be highly valuable; increasing egg production only upon mating would seem to be an efficient strategy to prevent unnecessary energy expenditure when males, or sperm, are not available for fertilization. Other examples might be less clear, like the reduction of female remating. While this is obviously advantageous for males to ensure the use of their sperm over that of their competitors, it seems less advantageous for females who might want to have the ability to trade up from a first sexual partner, leading to a potential case of sexual conflict^17^. That being said, there are other known examples of organisms that share this PMR with *D. melanogaster* despite the absence of *SP*. For example, the ground beetle females (*Leptocarabus procerulus*) are also refractory to remating like females *D. melanogaster*^18^ even though this species diverged from the *Drosophilinae* long before the emergence of *SP*^19^.

We have recently become interested in the species *Drosophila hydei*, a species that is part of the *repleta* group (*Siphlodora* subgenus) that diverged from *D. melanogaster* (S*ophophora* subgenus) about 60 million years ago^20^. Like *D. melanogaster, D. hydei* is a relatively common species, often found together with *D. melanogaster* in the wild^21,22^. Despite their relative environmental compatibility, the reproductive biology of the two species displays a few distinctive features. First, *D. hydei* males (along with other species closely related to *D. hydei*) have evolved unusually long sperm, often measuring over 2.3 cm in length (about 10 times longer than those of *D. melanogaster*)^23^. This odd sexual ornamentation seems to have had significant consequences on their reproductive strategy, being associated with a delay to the onset of male reproductive maturity (*D. hydei* males take approximately 10 days to reach sexual maturity vs 3–5 days for *D. melanogaster*) and limiting the number of sperm transferred to the female during mating^24,25^. A second significant difference from *D. melanogaster* is the loss of the *SP* gene. Recent work has shown that although *SP* exists in the Siphlodora subgenus, *D. hydei* is part of the *repleta/nannoptera* radiation, a branch of Siphlodora that has lost the *SP* gene^19^. Consistent with the absence of *SP*, in both *D. hydei* and *Drosophila bifurca*, at least one phenotype associated with SP activity in *D. melanogaster* is not found*;* indeed, mated females of both species tend to be willing to remate many times over a short span of time^26,27^. These fundamental differences make *D. hydei* an interesting choice to investigate PMRs and reproductive strategies beyond the *D. melanogaster* model.

Our investigations show that, unlike *D. melanogaster*, females *D. hydei* do not seem to produce more eggs after mating, as they are found to lay an equal number of eggs over a 3-day period regardless of mating status. Interestingly, mating seems to trigger only a burst of egg-laying that lasts for about 24 h, followed by a period of reduced egg-laying. In *D. melanogaster*, mating has also been shown to reduce female lifespan. Numerous factors have been suggested to be the cause for this reduction of lifespan, from direct signaling from male seminal fluid components like SP^28^, to the metabolic shift required for females to increase egg production^29^. As *D. hydei* do not possess *SP* and lay a similar number of eggs regardless of mating status, it was notable that we found that *D. hydei* females that intermittently mated to males lived equally long lives as their virgin female siblings. Video-assisted activity monitoring was then used to examine the activity patterns of mated *D. hydei*. Using this method, we were able to uncover post-mating changes in the *D. hydei* female circadian activity as well as other behavioral changes. Lastly, we investigated sperm competition in *D. hydei* and how changes in accessory gland structure seem to modify sperm competitiveness. Overall, our results show that *D. hydei* seems to have adopted a reproductive strategy that includes a set of previously uncharacterized PMRs that differ from those identified in *D. melanogaster*.

## Results and discussion

### Mated females show a short-term egg laying burst with minor increase in egg production in *D. hydei*

**Fig. 1:**
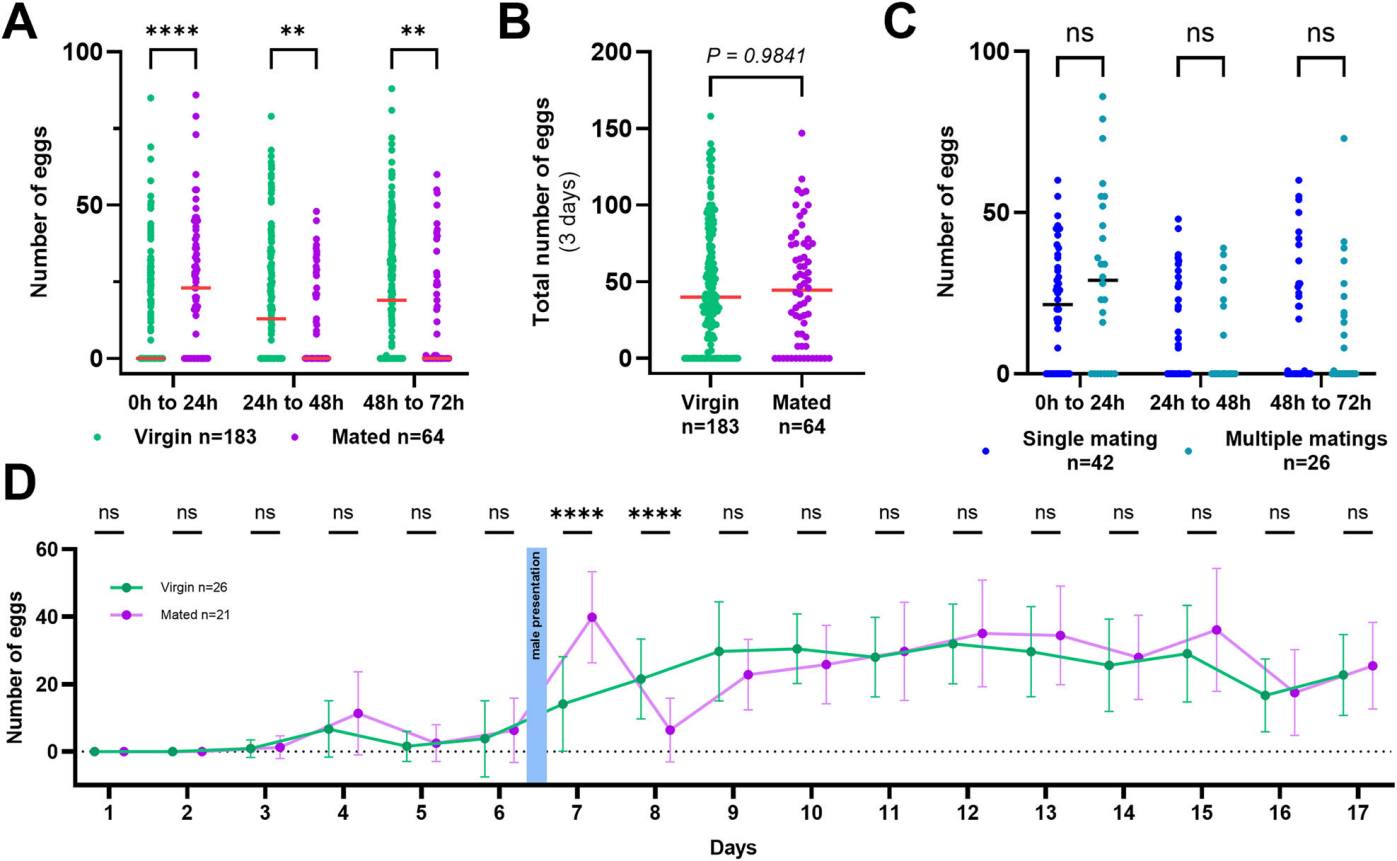
Egg laying in *D. hydei*. **A** Average number of eggs laid each day mated females during the 3 days post-mating or in virgin females of the same age. Mated females are females that have mated either a single time, or more than once in a rapid succession. **B** Total number of eggs laid by mated females for 3 days post-mating or by virgin females of the same age. **C** Comparison of the number of eggs laid by mated females in the three first days post-mating, after either a single mating event or a rapid succession of two or three mating events. **D** Average number of eggs laid by individual females throughout the 17 days after eclosion. Flies represented by the green line were mated on day 6 to males aged for 10 days. The total amount of eggs for 3 days (**B**) was analyzed using a test of Mann–Whitney. Daily egg laying data (**A**, **C**, **D**) were analyzed using a Mixed-effect model, for each day, conditions were compared using Šídák’s multiple comparisons test. ns (*P* > 0.05), ^*^(*P* ≤ 0.01), ^**^(*P* ≤ 0.01), ^***^(*P* ≤ 0.001), ^****^(*P* ≤ 0.0001).

Most of the best characterized PMRs in insects have been studied in *D. melanogaster*, where they are often linked to the SFP SP. To identify possible PMRs in a species without *SP*, we focused on *Drosophila hydei*. We first investigated the female’s egg-laying response. For this assay, we allowed individual females to mate freely over a 1-h period and then compared their egg-laying response to the number of eggs laid by isolated virgin females of similar age. Our results show that mated females lay more eggs than virgin females for the first 24 h post mating, but then begin to lay eggs at a lower rate than virgin for the following 24 to 48 h (Fig. 1A, D). After this short-term response in post-mating egg laying, the egg laying behavior of mated females returns to a level identical to their virgin counterparts (Fig. 1D), even though females will often continue to lay fertilized eggs from stored sperm. In some ways, this is similar to the response found in *D. melanogaster*. However, while in *D. melanogaster*, female egg laying is increased over a period of up to 10 days^30^, mated *D. hydei* females are found to lay a similar total number of eggs as virgins females over a 3 day period (Fig. 1B). This suggests a short-term response in the ovipositioning of stored eggs that seems to not be accompanied by an acceleration of egg production. In *D. melanogaster*, increase ovipositioning has been linked to SFPs like Ovulin and Acp36DE^11,12^. Interestingly, these proteins do not seem to be conserved outside of the *melanogaster* group^13^, indicating that the increase in ovipositioning in *D. hydei* must be induced via a different molecule/mechanism.

To confirm that the increased egg-laying rate observed in mated females results specifically from copulation, rather than male presence or courtship behavior, we compared isolated subjects against those paired with a male where mating did not occur for the duration of the experiment (Supplementary Fig. 1). As no significant difference could be identified between the two conditions, all of the virgin females were therefore used in the comparisons with mated females.

Based on our daily counting of eggs from virgin females, we find that virgins do not lay the eggs at a constant daily rate, but instead seem to store eggs for a period of time before “discarding” many eggs over a short period of time. This strategy means that a female will usually have a stock of eggs to use upon sperm transfer (consistent with the observed uptick in laying in the first 24 h after mating), but also that it might occasionally have only a few eggs to lay. As *D. hydei* males tend to transfer much fewer sperm^25^, this risk may be of lesser importance in the wild, unless multiple matings allows more sperm to be stored. As mentioned above, *D. hydei* females often mate multiple times, even during a typical mating assay. Thus, we decided to check if the number of matings influenced the number of eggs laid by comparing the number of eggs laid by females that mated once to those that mated twice or more times over the course of 2 h. Looking at the first 3 days post mating, we could not observe any significant difference between the two mating conditions (Fig. 1C). Thus, we find that rapid remating does not seem to result in the induction of a stronger egg-laying response. It is relevant to note that females in this assay were given the opportunity to remate with the same male multiple times, rather than with fresh males, as male *D. hydei* have been found to be capable of maintaining an almost constant number of sperm transferred when mated repeatedly^24,31^. Based on these results, it may be that *D. hydei* females benefit from the rapid response possible from constant egg production, especially if, in their natural habitat, mating possibilities are not limiting.

### Study of the impact of egg-laying on the lifespan of females

**Fig. 2:**
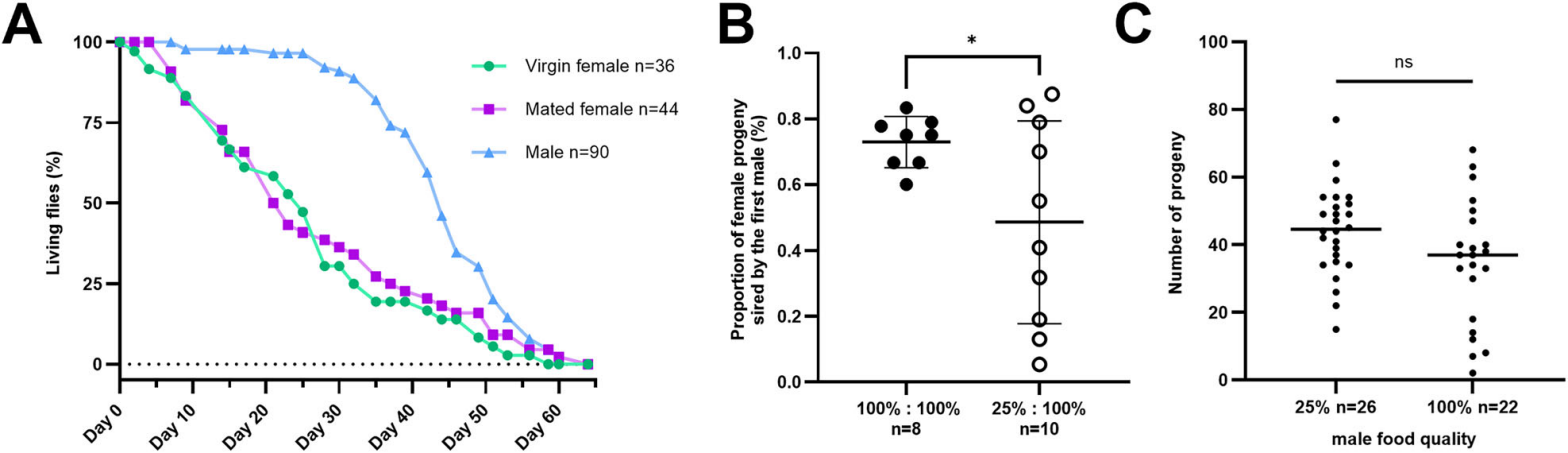
The impacts of constant egg-laying and multiple matings on survival and sperm competition. **A** Survival analysis of virgin females (green), mated females (magenta), and virgin males (blue) plotted as the percentage of total flies alive each day (Probability of Survival). Kaplan–Meier (with Mantel–Cox and Gehan–Breslow Wilcoxon tests) analysis shows no significant difference between virgin and mated females but significant differences between both female populations and males (*P* < 0.0001). **B** Sperm competition assay between males reared during all larval development on normal food (100%) or 25% diluted food (25%). Paternity of the female progeny could be determined by counting the number of w- or w+ females. The growth conditions of the males are listed on the *x*-axis; the first male growth condition is listed before the colon and the second male growth condition is listed after the colon (* indicates that there is a significant difference between the two experimental set-ups, *P* = −0.0461, Kolmogorov–Smirnov test). **C** Total progeny from singly mated females from males grown under each growth condition. No significant difference between each condition was found (Mann–Whitney U-test *P* > 0.05).

In *D. melanogaster*, the massive induction of egg production necessitates dramatic changes in the female physiology that seem to have costs on the female body^28,32^, leading to a decrease in lifespan^33,34^. As *D. hydei* females seem to produce eggs at a constant rate, we wondered if mating might still result in female lifespan reduction. To test this, we performed survival assays on both mated and virgin *D. hydei* females as well as virgin males. For these experiments, we housed flies in groups of approximately 10 flies per tube. For the mated female group, once a week, for 3 weeks (skipping the first week due to female immaturity), we added an equal number of fertile males and allowed mating overnight before removing the male flies. Using this protocol, we find that virgin and mated females survive similar times (and less than their male siblings) (Fig. 2A). Interestingly, in a separate experiment, we examined the survival of females and males housed together. In this experiment, females housed with males showed a significantly lower lifespan than virgin females. Thus, it seems that constant exposure to males, or frequent mating events, may have a negative effect on *D. hydei* females that is not seen by intermittent matings (Supplementary Fig. 2). This latter trend is similar to that observed in *D. melanogaster*, where females undergoing intermittent matings lived longer than females continuously exposed to males^33,35^.

### Male health and sperm competition

As mentioned above, we and others found that *D. hydei* females often mate multiple times during the timeframe of a mating assay. These matings are relatively short, generally lasting under 3 min. However, our data shows that multiple matings over this short timeframe do not seem to yield a statistical increase in the number of progeny produced (Fig. 1C). Thus, we wondered why multiple mating may have been selected in *D. hydei*. One possible answer was that multiple matings might be a mechanism to allow females to select better sperm and may actually be linked to the sexual selection for longer sperm in this species. In order to investigate this, we first performed sperm competition assays by crossing *white* mutant *D. hydei* females to either *white* mutant males or *wt* males, followed by immediately crossing these singly-mated females to second males of the alternate genotype (*w*^*+*^ or *w*). When the progeny of these crosses eclosed, we were able to score the eye color of the resulting female progeny to determine the male whose sperm was used by the female to fertilize her eggs. In *D. melanogaster*, it is generally found that when secondary matings occur, there is a last male precedence, where the sperm of the last male copulating is used over that of previous matings^36^. In *D. hydei*, we find the opposite to be mostly true, in that the first male’s sperm seem to produce more offspring than that of the subsequent male (Fig. 2B). This result corroborates previous characterization of *D. hydei* as a species where sperm of subsequent males are not preferred over the precedent^31^.

We then asked if this ratio could be modified based on the health of the male. As our hypothesis was that this may be a method for the female to select sperm from healthier males, we sought out treatments that might subtly affect the male health. Previous studies have shown that the nutritional environment under which flies develop affects male size and fertility^37^. Interestingly, in *D. melanogaster*, the reduction in male fertility may be independent of the individual sperm cells themselves, as sperm length seems to be similar between nutritionally minute flies and control flies^37^.

In our sperm competition assay, we tested the ability of flies grown under reduced nutritional conditions (grown on 25% nutrient food) to defend against secondary sperm coming from a fly grown under standard conditions. Measuring the thorax size of flies grown under each condition confirmed that flies grown on 25% food were smaller than flies grown under standard conditions (Supplementary Fig. 3A). To see if sperm size changed in these two populations, we then measured the length of the testes of flies grown under the two nutritional regimes (Supplementary Fig. 3B). As testes length positively correlates to sperm length in flies and is much easier to measure than the tangled, multiple-centimeter long sperm of *D. hydei*, we, like others, decided to use this as a reasonable proxy for sperm length measurements^38^. Based on the similarity of testes lengths, we believe that, like in *D. melanogaster*, sperm length is not affected by our manipulations^37^. In competition, however, we find that sperm from nutritionally starved males (25% food) were less able to compete with the sperm of control males. As seen in Fig. 2B, control males show a first male preference. However, this is reduced when the first male was raised on lower-quality food. As starvational changes in the male might cause the female to be less receptive, we verified all matings by visually monitoring all matings. We thus hypothesized that this might be due to a reduction in sperm transfer, as others have reported that minute *D. melanogaster* males make and transfer fewer sperm than wild-type males^39^. However, this effect was only visible through a cumulative effect from multiple matings by the undernourished males^39^. Our results indicate that females mating once to either type of virgin male produces a similar number of offspring (Fig. 2C), suggesting that a similar number of sperm are transferred from virgin males of each condition after a single mating to a virgin female.

### Variation in the shape and structure of the male accessory gland does not correlate with the loss of *SP*

Assuming the sperm component of the seminal fluid is not changed (in length or amount transferred), we hypothesized that other seminal fluid components might influence the sperm competition. In *D. melanogaster*, this is known to be the case, as SP and the proteins that associate SP to sperm are known to effect sperm competition^12,40–43^. As these proteins are known to be affected by the mAG, we examined the effect of food reduction on mAG growth. We started this by first examining the mAGs of control flies from *D. melanogaster* and *D. hydei*. In well-nourished *D. melanogaster* males, the mAG consists of a pair of sac-like lobes that are approximately 800 µm in length that are made up of two secretory cell types. The main cells that form most of the gland and the secondary cells (SCs), which represent around 40–50 cells per lobe, and are located at the distal tip of the gland, interspersed with main cells. The SCs are larger than the main cells and are filled with characteristic, large vacuole-like structures^44^.

**Fig. 3:**
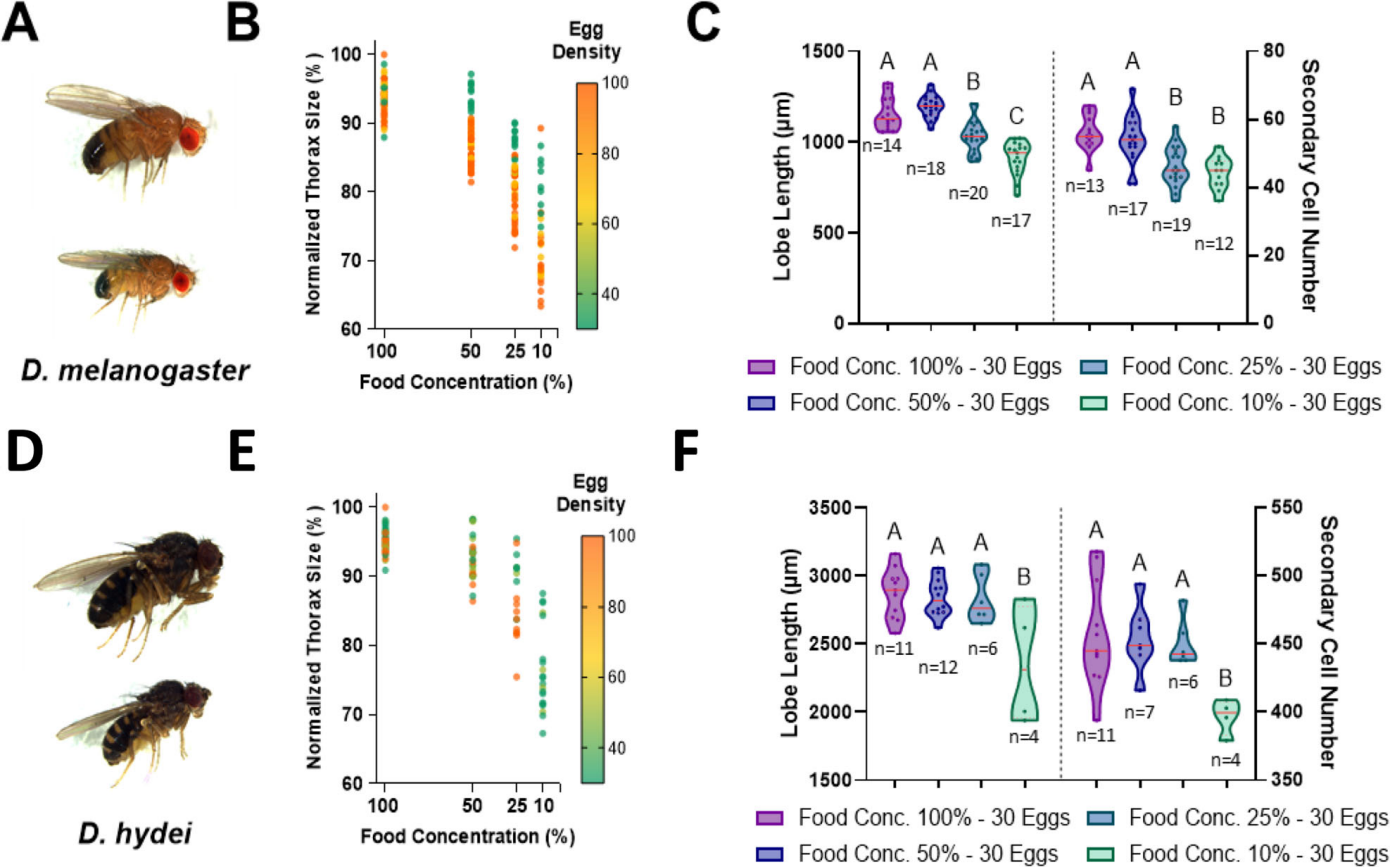
Thorax size and accessory gland morphology under starved developmental conditions. The species used were *D. melanogaster (top), and D. hydei (bottom*). **A**, **D** Comparison of the flies’ size, at the same scale, between the most concentrated food (100%) and the least (10%). **B**, **E** Thoracic size is represented as a ratio of the average size of the thoraces relative to the largest size of thoraces of flies reared on 100% food. The concentration of the food they’ve been reared on (*x*-axis), and the density of the eggs that were put in the tube (color). **C**, **F** Measurement of the size of the gland’s lobe and the number of SCs they contain on different food concentrations. Analyzed using ordinary one-way ANOVA.

To investigate if our food manipulation might have the potential to change the composition of the seminal fluid, we examined the size and structure of the mAG after growth on different media concentrations. Previously, we used a single treatment condition, where approximately 70–100 embryos were transferred into large tubes containing about 8 ml of food that was diluted to 25% of its normal nutrient concentrations. For a more precise measurement of the effect of the nutritional composition of our media on mAG structure, we placed precise numbers of embryos (*D. melanogaster* or *D. hydei*) into small tubes containing 3 ml of food diluted to four different food concentrations (100%, 50%, 25% and 10%). Upon eclosure, males were collected and transferred to normal food tubes to mature (5 days for *D. melanogaster* and 10 days for *D. hydei*). The thorax of some of these males was measured to confirm the effect of reduced nutrition on the adult flies. For some combinations of high egg density and low food concentrations, surviving adults could not be isolated. For this reason, we chose to dissect males from the 30 egg count density because adult flies could be obtained at all food concentrations. Dissection of these males showed that the accessory gland size and the number of SCs were affected by food quality, particularly at lower food concentrations (Fig. 3).

In *D. melanogaster*, the function of the SCs seems to be tightly linked to the protein SP. Defects in the SCs lead to a phenotype where the PMR does not last for more than about 24 h^30^. This defect in the so-called long-term PMR is due to SP failing to be attached to sperm as they enter the sperm storage organ. We now know that SCs produce, among many other proteins, many of the proteins that are necessary to attach SP to sperm, allowing it to be slowly released over the following days and continually induce the SP response^45^. Interestingly, *D. hydei*, which lack the *SP* gene, have about 10× the number of SCs as *D. melanogaster* (Fig. 3). This suggests that SCs may play important roles outside of attaching SP to sperm. We decided to explore the relationship between the *SP* gene and mAG structure by examining the mAGs of different sequenced *Drosophila* species from across the *Drosophila* phylogenetic tree.

**Fig. 4:**
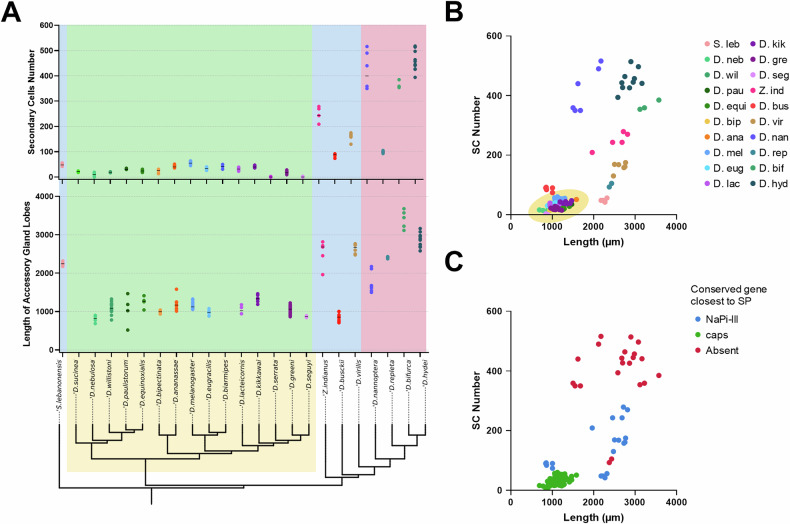
Accessory gland lobe length and secondary cell numbers in Drosophila species. **A** Phylogenic distribution of Secondary Cell number (top) and Lobe length (bottom). The Sophophora subgenus is highlighted in yellow on the phylogenetic tree. The location of the SP gene is indicated with background coloring: green – near caps; blue, near NaPi-III, red – absent. Phylogenetic tree based on ref.^14^. **B** Number of SCs relative to the length of the lobes, colored per species. Species that are part of the Sophophora subgenus are highlighted in yellow. **C** Number of SCs relative to the length of the lobes, colored in respect to the position of the SP gene.

We first examined flies in the *Sophophora* subgenus. In this radiation, the *SP* gene has been shown to be highly conserved and has even undergone gene duplications^19^. This subfamily is also characterized by the *SP* gene being translocated to a different location, near the gene *capricious* (*caps*), from its ancestral location, near the gene *NaPI-III*^14^. For example, *D. melanogaster* has two copies of a SP-like gene near *caps*, while some species in the *ananassae* group have as many as 7 copies. Within the flies examined from the *Sophophora* subgenus, all had glands with relatively small lobes reaching no more than 2000 µm in length, generally between 1000 and 1500 µm, like in *D. melanogaster* (Fig. 4A, B, *yellow highlight*). Furthermore, we found that the glands rarely contained more than 50 SCs per lobe, with some, like *Drosophila serrata*, having few or no SCs (Fig. 4). In this subgenus, the SCs present were always located at the distal tip of the lobes (Fig. 4A, B, and Supplementary Fig. 4).

When examining the mAGs of non-*Sophophora* species (Fig. 4A, B), glands tended to be longer, reaching up to 3000 µm in length. Besides the length, we also found that SCs were often spread across the entire length of the lobes (Supplementary Fig. 4), rather than sequestered at one end. The number of SCs in these species was highly variable, from around 150 SCs per lobe in *Drosophila busckii* (Fig. 4A, B) to more than 400 in *Drosophila nannoptera* and up to 500 in *D. hydei* (Fig. 4A, B). Interestingly, the dichotomy between species that have short glands with few SCs and those that have long glands with many SCs coincides with the divergence of the *Sophophora* group.

We also examined the glands of two more basal species. Surprisingly, the glands of *Scaptodrosophila lebanonensis* are long, but with SCs located only in the center of the lobes (Supplementary Fig. 4). Another species, *Chymomyza pararufithorax* (Supplementary Fig. 5), also has long glands and has many (but uncounted due to the difficulty in culturing these flies) SCs spanning about ¾ of the gland from the distal tip. Based on these results, it seems likely that ancestral mAGs were probably more similar to what is found in the non*-Sophophora Drosophila*.

We attempted to understand if changes in the mAG structure could manifest in changes to the PMRs by performing 3-day egg laying assays for a number of species (Supplementary Fig. 6). These assays, while not conclusive, hint that each of these species may have slightly different PMRs regarding egg laying/production. For example, our data indicates that certain species (*Drosophila paulistorum, Drosophila ananassae* and, to some extent, *Zaprionus. indianus*) have a delayed, but longer-lasting egg laying response after mating. These particular species all have the *SP* gene, with *D. ananassae* seemingly having additional gene duplications from *D. melanogaster*^19^. This may indicate that females of these species mature their eggs only after mating occurs, though more work would be required to confirm this. Many of the other species seem to have a short-term boost in egg-laying that is mostly seen in the first 24 to 48 h post mating. Given the link between SCs and the long-term PMR identified in *D. melanogaster*, it is interesting that we found relatively short-term responses in species like *D. virilis* that has *SP* and more than 150 SCs, and a relatively long-lasting PMR in *D. serrata* that also has *SP*, but seems to have almost no SCs (Fig. 4, and Supplementary Fig. 6).

The *SP* gene seems to have first appeared in the *Chymomyza* lineage^14^. Our finding of numerous SCs in a species of this group (*C. pararufithorax*) suggests that the presence of *SP* was probably not a prerequisite to having SCs and that SCs likely played a different role in male reproduction prior to its role in SP attachment. Meanwhile, the repeated loss of *SP* in drosophilids that still have SCs, like *D. hydei*^19^, indicates that these functions may still be present. Due to the link between mAGs and seminal fluid production, it seems likely that changes in male nutritional status could affect the non-sperm constituents of the seminal fluid. As SC proteins in particular seem to somehow help attach proteins to sperm, we might hypothesize that longer sperm might require more SCs. In support of this, species in the *repleta/nannoptera* radiation tend to have longer sperm tails than *Sophophora* species. *D. hydei*, for example, has sperm of ~2.3 cm^24^ and contains hundreds of SCs, while *D. melanogaster* has sperm of less than 2 mm^24^ and has only ~40 SCs.

*D. hydei* and its related species have long been an enigma to evolutionary biologist because of their extremely long sperm^37^. Longer sperm require more time and energy to produce^46^. As a consequence, these long-sperm species tend to mature slower after eclosion^24^, be of larger size^23^ and make fewer sperm when compared to shorter sperm species^37,46^. Given that it has been shown that increased fertility seems to be linked to producing more sperm^47^, it has been a mystery as to why females tend to select longer sperm vs shorter sperm, when her sperm storage organs can accommodate them^48^. One hypothesis to explain this enigma is that long sperm might represent a form of sexual ornamentation to allow females to potentially judge the quality of males. Indeed, nutritionally deprived males seem to be less competitive in sperm competition assays. However, we, as have others, do not see a change in sperm length after nutritional deprivation^37^. Given the changes we document in the mAG associated with nutritional deprivation, and the connection of SC products to sperm modification (protein attachment), it is interesting to hypothesize that mAG products attached to sperm might allow for the selection of longer sperm and healthier males through the amount of these products attached.

### Mating induces long-term changes in a highly stereotypical circadian activity

**Fig. 5:**
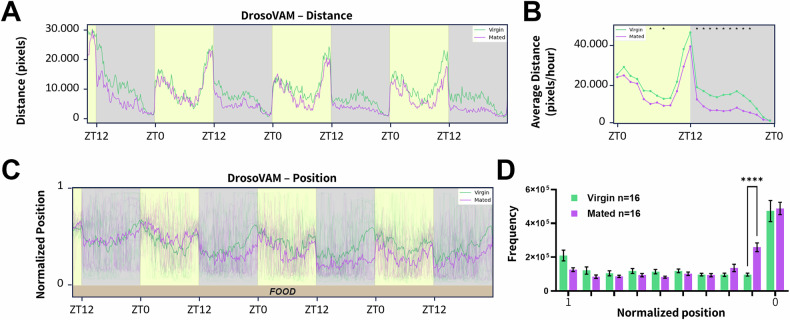
Analysis of the circadian activity of mated and virgin *D. hydei*. Light/Dark periods are shown with yellow/grey background, respectively. **A** Distance in pixels moved by mated and virgin females for 86 h post-male presentation, bins of 10 min, *n* = 16/16. **B** Average Day of the distance moved by mated and virgin female over 24 h. Average day is composed of three days of monitoring. Statistical analysis was performed using tests of Mann–Whitney (* = *P*-value < 0.05). **C** Temporal representation of the normalized position of the flies in the locomotor activity chambers. The extremity of the chamber in which food is present is located at the bottom of the chart. Positions of all the flies are shown (transparent lines) and the average position is represented with the solid line, bins of 10 min. **D** Histogram of the position of the flies in the DrosoVAM chamber over 86 h. The side of the chamber where food is located is represented on the right of the histogram. Statistical analysis was performed using ANOVA2 (“Position X Mating Status” *P*-value < 0.0001), followed by Šídák’s multiple comparisons test (^****^P-value < 0.0001).

To uncover other PMRs in *D. hydei*, we decided to examine some of the known behavioral responses of *D. melanogaster* or other species. Previously, it has been reported that there is an increase in daytime locomotor activity in *D. melanogaster*, with virgin females displaying a stronger daytime sleep behavior^49^. Furthermore, it has been reported that there is a relative decrease in the activity of mated females just prior to circadian lights-on (morning anticipation, more pronounced in virgins)^7^. Analyzing the locomotor activity of virgin *D. hydei* females using the Drosophila Video-assisted Activity Monitor system (DrosoVAM)^50^, we were able to document a highly stereotypical activity pattern in *D. hydei* with clear peaks of activity in the morning and evening (Fig. 5A). Although at a cursory glance, this pattern seems similar to that of *D. melanogaster*, it shows no sign of a classical morning anticipation. Indeed, *D. melanogaster* virgin females display an increase in activity as the time approaches circadian dawn/lights-on, while the activity of *D. hydei* increases only after the light turns on. Under constant darkness conditions, these sharp peaks of activity during the day, particularly at lights-on slightly fade, indicating that it is mostly a startle response to the light (Supplementary Fig. 7A). Nevertheless, we do see an overall increase in activity during the day and an alternative kind of morning anticipation, characterized by a pre-dawn static period where activity drops for the last 2 to 3 h before lights-on (Fig. 5A, B). These activity trends are found in both virgin and mated females, as well as in males (Supplementary Fig. 7B). When examining the differences between mated and virgin females, we noticed that mated females seem to be less active overall, with the effect being more notice during the nighttime hours. Averaging the distance moved for each hour of the day over three and a half days, we see that mated females move significantly less than virgins for a few hours in the middle of the daylight period and also for most of the night (Fig. 5B).

Comparing virgin and mated locomotor activity over several days post-mating suggested an overall reduction of the activity in mated females (Fig. 5A, B). Interestingly, this reduction in movements was found to be more important at night, and to be maintained for more than 72 h after mating. To understand why mated flies move less than virgins, we decided to look at the position of the flies. As DrosoVAM is based on video tracking, we could plot the position of flies over time relative to the food source. We identified that around 36 h after mating, mated females start to stay closer to the food than their virgin counterparts (Fig. 5C). This effect could be confirmed by examining the overall position of the flies over the duration of the monitoring, showing that mated females are more commonly found on or near the food relative to virgin flies (Fig. 5D).

Several reasons could explain the preference to stay on/near the food for mated females: the need to lay eggs, and the need to eat more. Egg laying has been found, in isolated *D. melanogaster* to be happening preferentially in the dark^51^, which may explain the need to move closer to a food source at night. Interestingly, using the same methodology, we found that males also tend to stay further away from the food than females (Supplementary Fig. 7C). Thus, another possibility for mated female relocation could be that this relocation is a way for the mated females to partition themselves away from males for one reason or another.

### Mating triggers a shift in light preference but not in food source preference

**Fig. 6:**
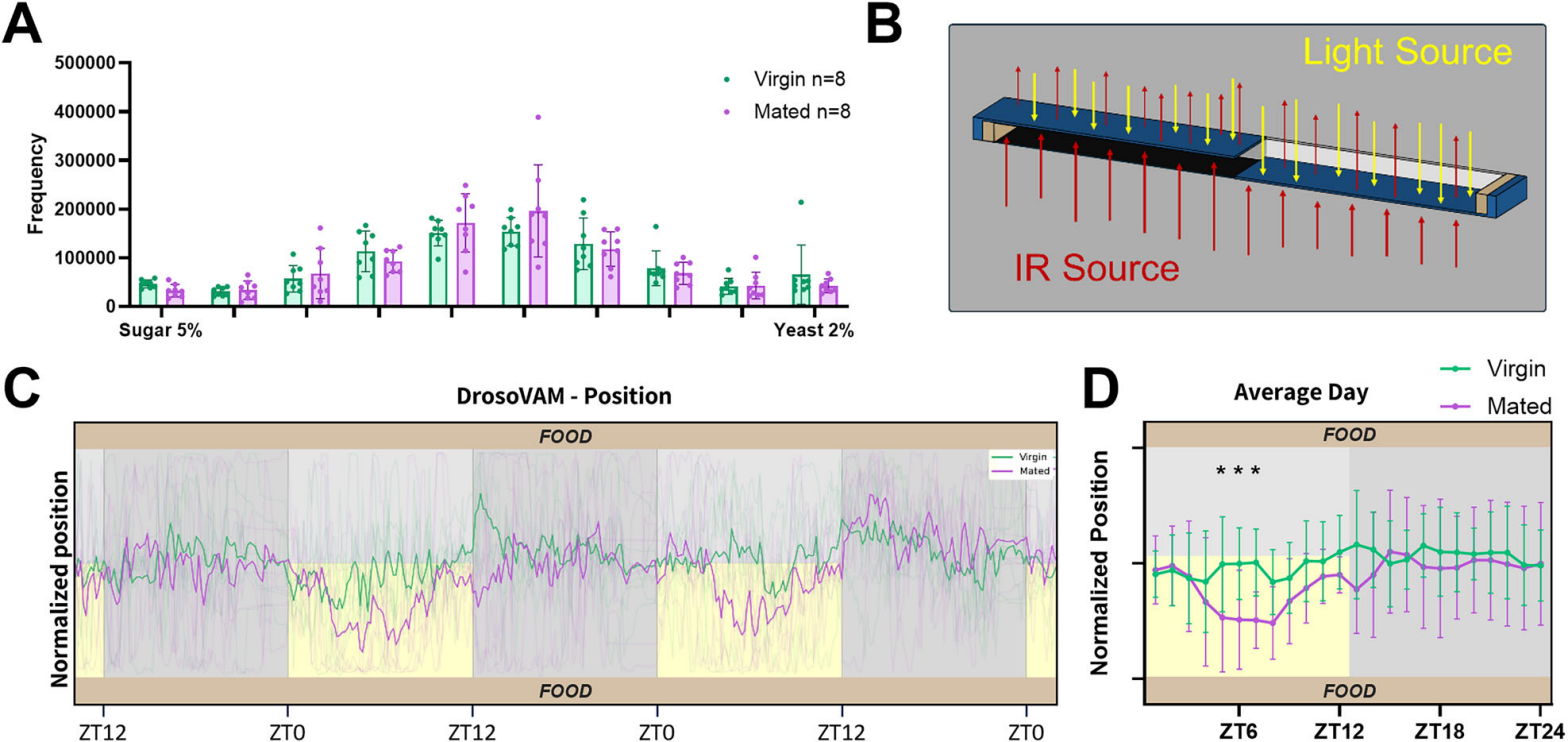
Position of the flies along the food/ light preference chambers. **A** Histogram of the position of the flies along the food preference chambers. The chambers are divided into 10 bins (~5 mm per bin) and the amount of time each fly could be found in that position over 24 h was quantified. The side of the chamber with yeast-containing food is placed to the right while the side of the chamber with a simple sugar agar solution is placed to the left. *T*-tests performed between the positions closest to the food and between virgin and mated females (*n* = 16/16) and found no significant difference. **B** Design of a single Darkness Preference Chamber. The basic shape of the chamber is the same as for a food preference assay. A filter film, mostly transparent to infrared (IR) light, but opaque to visible light was applied to both sides of the chamber. On one half, the filter was placed between the light source and the chamber, while on the other half, it was placed between the IR source and the chamber. This second filter, below the chamber, was necessary because of the slight reduction of the IR light transmitted to the camera (from below the chamber relative to the camera). Without the two filters, tracking quality decreased as contrast between the two sides of the chamber was vastly different. In these chambers, identical food sources are placed at both ends. **C** Temporal representation of the position of the females in the darkness-preference assay chambers. Positions of all the flies are shown (transparent lines) and average position is represented with the solid line. **D** Average position of flies at each hour of the day over the course of the experiment. Statistical analysis was performed using ANOVA2 (“Time X Mating Status” *P*-value = 0.0211), followed by Šídák’s multiple comparisons test (*P*-values: ZT5 = 0.0477, ZT6 = 0.0294, ZT7 = 0.0216).

In *D. melanogaster*, Sex Peptide has been found to trigger a shift in food source preference in mated females; mated females show a preference for yeast over sugar^52,53^. Such a food preference assay can be performed using DrosoVAM by examining fly positions in chambers with two food sources. For this assay, we tested mated female *D. hydei* in chambers with either an agar mix complemented with 2% yeast or 5% sugar, a condition that we previously showed to work for mated *D. melanogaster* females^50^. However, when using this assay for *D. hydei*, we could not identify any food preference in virgin or mated flies (Fig. 6A).

Because of the differences that we found between night and day activity and the localization of mated females, we wondered if the changes could be linked to light exposure. Taking advantage of the versatility of the DrosoVAM system, we decided to transform the food preference chambers into darkness preference chambers to evaluate if mated flies would change their light preference relative to virgins. For these assays, we used filters, opaque to visible light but not infrared, to shade one half of each chamber with identical food sources at both ends (Fig. 6B). Interestingly, in this assay, we observed a slight but significant preference of the mated females for staying in the non-shaded part of the chamber during lighted hours, whereas virgin flies displayed no preference (Fig. 6C, D). Meanwhile, it seems that males mostly prefer to avoid the light during the day (Supplementary Fig. 8).

In the wild, most *Drosophila* have been reported to be found in shaded areas during the daylight hours more than in full light^54^. Thus, it is puzzling to find that mated females seem to preferentially move towards the light side of our light preference chamber, as it is assumed that being in lighted areas would place flies at increased risk of predation. One potential hypothesis to explain this unexpected phenotype is experimental. As both sides of the chamber were made to be as similar as possible except for the IR-pass filter, it is possible that there might be a slight temperature difference between the two sides of the chamber. In order to equalize the video lighting from our IR camera, we place filters on both sides of the chamber with one side of the chamber having the filter between the fly and the IR source and the other half of the chamber having the filter between the fly and the visible light source and camera (Fig. 6B). As IR light is associated with thermal radiation, it may be that there is a slight temperature difference between the two sides of the chamber, with the filter potentially cutting some of the IR light. This would mean that the portion of the chamber unshaded to visible light would be a bit cooler than the shaded side. This effect would be constant as the IR light source is on during the whole day/night cycle. Thus, if temperature is the reason behind the behavior observed, it means that the PMR is actually that mated females prefer cooler areas during the day and not at night. Yet, if this laboratory phenotype has been interpreted correctly and mated females prefer lighted areas over shaded, then we must conclude that the benefits of spending more time in the light outweigh the risks or that the assumed risks are overestimated. One possible benefit of this light preference could be the creation of a location-based separation from males to allow females to perform activities without the constant courtship from males, who prefer to stay out of plain light. This would provide a similar function to the SP-induced refractivity towards remating that is seen in *D. melanogaster*. Interestingly, other Drosophila species have also shown novel mate-guarding phenotypes. For example, *D. pegasea* and *D. mainlandi*, two other species without *SP* were found to have independently evolved a mate-guarding mechanism where the male is found riding the back of the female for hours after initial mating completion^55,56^. In a similar fashion, *D. acanthoptera*, which also lacks *SP*, was found to maintain mating for more than 3 h in experiments conducted in the lab, which could be another form of a mate guarding strategy. Further examination of *D. hydei* in a more natural setting would allow for a much better interpretation of our results.

### Outlook on the reproductive strategy of *D. hydei*

Although *D. hydei* can often be found living along *D. melanogaster*, their PMRs are very different. Much of this may be linked to the evolutive selection for long sperm vs increased brood size. In *D. melanogaster*, males produce and deposit hundreds of sperm in a single mating^57^. To utilize these sperm, *D. melanogaster* females must produce a proportional number of eggs, an energetically costly endeavor that requires changes to the female metabolism and a lowering of her overall fitness^58,59^ Given the costs of egg production, it seems beneficial for *D. melanogaster* females to maximize egg production only when fertile males are present. Using a SFP, like SP, to trigger this increased production might be preferable to mating-independent mechanisms that would be detrimental to the female when fertile males are not present. In contrast, *D. hydei* males produce and deposit less sperm^24,25^. Work performed in *D. bifurca*, a related species with extremely long sperm, has correlated the decreased sperm transfer and a decreased number of eggs produced^26^. Here, we find evidence suggesting that *D. hydei* females produce a similar number of eggs regardless of mating status. As repeated matings over a short period of time do not seem to increase the total number of eggs laid or progeny sired, it appears that *D. hydei* females receive sufficient quantities of sperm from a single mating to fill their storage organs and allow them to fertilize eggs for 2 to 3 days post mating. In the natural environment in which *D. hydei* reproduce, perhaps it is beneficial (or at least not detrimental) for a female to keep a stable metabolism, which would preserve her overall fitness, while selecting for sperm quality over quantity. Additionally, due to the limited number of sperm transferred, investment into a system to trigger a high level of egg production may not be sufficiently advantageous. It is worth noting that many mated behaviors in *D. melanogaster*, like increase egg laying and refractoriness, seem to be the default neurogenetic state, which is suppressed in mature virgin females^60^. In *D. hydei*, perhaps this repressing system has simply been lost along with the evolution of longer sperm, therefore removing the need for the male ejaculate to lift of this inhibition. Alternatively, it is possible that such a repressing virgin-state mechanism has evolved specifically in species relying heavily on SP.

Overall, our study highlights that different reproductive strategies can be observed and be successful in nature, even within the *Drosophila* genus. Although the SP strategy has proven to be valuable to *D. melanogaster*, it is interesting to note that it may only be within the *D. melanogaster* species group that SP has taken on such an important function. Studies have shown that the injection of SP into species of the *D. melanogaster* group induces *D. melanogaster*-like PMRs, while similar injections of SP into species outside of the *D. melanogaster* group do not, even when injecting their own SP orthologs^16^. In fact, in this same study, *D. melanogaster* was shown to respond to SP orthologs from species outside of the *D. melanogaster* group, demonstrating the increased potency of the SP response in *D. melanogaster*^16^. The increased importance of SP in the *D. melanogaster* group may be linked to changes in the expression of its receptor, as SPR was also found at higher levels in the female reproductive tract of this group^16^. Alternatively, another hypothesis is that the central role of SP in this phylogenetic radiation can be linked with the identified changes in *SP* genomic location and the propensity of *SP* gene duplications within the *melanogaster* group^19^. Meanwhile, *D. hydei* and its related species seem to have chosen a different strategy, likely linked to increased sperm size. Although we and others have provided some evidence suggesting that this may be a method for females to select “better” males, its selective advantage is still not perfectly clear^37,61^. What seems clear, however, is that there are many solutions to the question of reproductive strategy and that these choices can have important consequences on the ecology of an organism. Only by studying other species can these strategies be discovered.

## Methods

### Drosophila strains

*D. hydei* strains were kindly provided by Benjamin Loppin (LBMC, ENS, Lyon, France); all other non-*melanogaster* species were kindly provided by Markus Knaden (nGICE, Max Planck Institute, Jena, Germany). All species were dissected at reproductive maturity upon arrival and/or housed in standard conditions at 25 °C on standard *D. melanogaster* cornmeal food.

### Dissection and imaging

For the accessory glands, males of each species are isolated from same-age stocks a few days after reaching sexual maturity and dissected in 0.3% Phosphate Buffer Saline (PBS) -Triton. Glands are isolated and transferred into 1 ml of 0.3% Triton-PBS. Glands are fixed by adding 110 µl of 36% Paraformaldehyde and incubating at room temperature for 10 min on rotating wheel. Without removing the PFA, glands are then stained by adding 120 µl of Ethidium Bromide at 1% and incubating for another 10 min at room temperature on a rotating wheel. All liquid is removed and the glands are washed twice for 10 min in 1 ml 0.3% PBS-Triton. Glands are then transferred onto a slide with Vectashield, covered with a cover slip and sealed with nail polish. Glands were imaged using the Ni-E confocal microscope from Nikon with the NIS-Element C imaging software. Images were then analyzed using Fiji (ImageJ2) to measure the glands, and Secondary cells were counted using the Cell Counter Fiji plugin (https://imagej.net/ij/plugins/cell-counter.html).

For imaging eggs, the eggs of the flies that were used in the malnourished vs nourished egg-laying assay were collected from the tubes and separated from the food in 1X PBS. The clean eggs were transferred onto a slide and fixed with 3.6% Paraformaldehyde. The slides were covered with a cover slip and sealed with nail polish. Imaging was done using Axioplan2 and Fiji (ImageJ2) was used to measure the length.

For measuring thorax and testes sizes, *D. hydei* males that were used in the sperm competition assays were dissected in 1X PBS. Their thorax and testes were isolated and transferred onto a slide and then fixed with 3.6% Paraformaldehyde. Thoraces were placed into a channel made by gluing stacks of coverslips to a slide. Space within the channel was filled with 10% glycerol and the whole area was covered with an additional coverslip and sealed. Testes were dissected and extended on slides in PBS, in a way that made measuring possible while trying to minimize tissue stretching. We measure testes in a way that includes the seminal vesicle due to the difficulty in judging exactly where each part began/ended. The slides were covered with a cover slip and sealed with nail polish. Imaging was done using Leica DM5500 B. Fiji (ImageJ2) was used to measure the size.

### Mating and egg laying assays

Each female was individualized a few days prior to mating and placed on a 12:12-h light:dark cycle. About 1 h before expected lights-on, ~10-day old male *D. hydei* of the appropriate genotype were added to each tube (diameter 13 mm) and visually monitored until mating occurred. Upon mating completion, the male was either allowed to remate with the female, in the case of multi-mating assays, or removed. For egg laying assays, virgin or mated females were then transferred to new tubes and left at 25 °C. Virgin females were those females that were presented males, but did not mate, or females kept in isolation. Virgin females of both types displayed similar egg-laying profiles and were therefore pooled into one group labeled virgin females (see Fig. S8). Tubes were then changed every 24 h, and the number of eggs was counted. Egg numbers were then entered into GraphPad PRISM (Version 10) for subsequent analysis and plotting.

For other species, experiments were performed in a similar manner. Both males and females were used when reaching sexual maturity, as previously characterized in the literature^24^, or as defined by experiments in the lab.

### Sperm competition assays

For sperm competition assays, a similar mating protocol was used as above. For these assays, w- female flies were used and mated to either w- or w+ flies depending on the experiment. All matings were monitored during the course of these experiments and only witnessed matings were scored. We performed the experiment in both directions and found that there was no change linked to the first male being w- or white+. Also, due to the overall lower mating rate in *D*. hydei, we performed these experiments over multiple days and combined all of the results.

In order to check for the influence of nutritional state on sperm competitiveness, we placed ~70–100 embryos from overnight egg layings of w+ or w- stocks onto tubes containing either 8 ml normal food (per 10 L standard food: 0.65% (w/v) agar, 6% (w/v) powdered yeast, 6% (w/v) corn meal, 7% (w/v) sugar, 0.3% (w/v) Nipagine, 1.7% (v/v) propionic acid) or an equal volume of normal food diluted 1:3 with 1% agar. Males were collected and aged in groups of 20–25 males for ~10 days in normal food. Mating was performed as above. Random males that mated were measured for their thorax size and their testes length (see above).

Female progeny were counted and scored for eye color. Due to occasional false mating, only tubes with both w- and w+ progeny were counted as having mated multiple matings

### Survival assays

For the intermittent mating survival assay, a total of 80 virgin females were placed in tubes in groups of eight or nine flies per tube. The 89 males were also divided into tubes in groups of eight or nine. At Day 6, an equal number of 10-day-old males were added to each 5 tubes, with the remaining tubes kept as virgins. Overall, 44 females were mated. And 36 females (less due to some flies escaping) were kept as virgins. Both a week and 2 weeks later, the matings were repeated. Flies were transferred every 2–3 days into fresh tubes, each time counting dead flies and removing them.

For a constant mating survival assay, 100 males and 96 females were kept as virgins, while 49 females were housed with 50 males. The flies were divided into tubes in groups of 20 of the same sex, and or approximately 10 females and 10 males. Tubes were changed every 2 to 3 days as above, and dead flies were counted.

### Locomotor activity, position analysis and preference assays

Males, virgin females, or mated females were loaded into 3D-printed chambers, recorded, tracked and analyzed as described in ref.^50^. To set up the darkness-preference assay, the food preference arena has been modified using an IR-pass plastic. Such plastic was obtained using the magnetic band of old floppy disks (diskette). IR-pass plastic was applied differentially on the upper- and lower-halves of the chambers. On the upper part, plastic was applied on the top of the chamber, blocking most of the visible light, and dimming only slightly IR light. The plastic was also applied on the bottom of the lower half of the chamber to not block any visible light, while still equally dimming IR light. The interface between the two halves of the chamber caused a slight alteration in the ability of DeepLabCut^62^ to detect flies crossing the interface but nevertheless allowed us to track the flies in the rest of the chambers.

### Reporting summary

Further information on research design is available in the Nature Portfolio Reporting Summary linked to this article.

## Supplementary information

Supplementary Information

Description of Additional Supplementary Files

Supplementary Data

Reporting Summary

Transparent Peer Review file

## Data Availability

All data used to create the figures in this manuscript are attached as Supplementary data (Revel_et_al_data-1-xlsx).
