# Supplementary material for: Characterization of the post-mating responses of Drosophila hydei, a species that lacks Sex-Peptide
Source: Commun Biol. 2026 Apr 11;9:865. doi: 10.1038/s42003-026-10021-5 (PMC13315242; doi:10.1038/s42003-026-10021-5)
Supplement: Supplementary file 1 — Supplementary Information [file 42003_2026_10021_MOESM1_ESM.pdf]

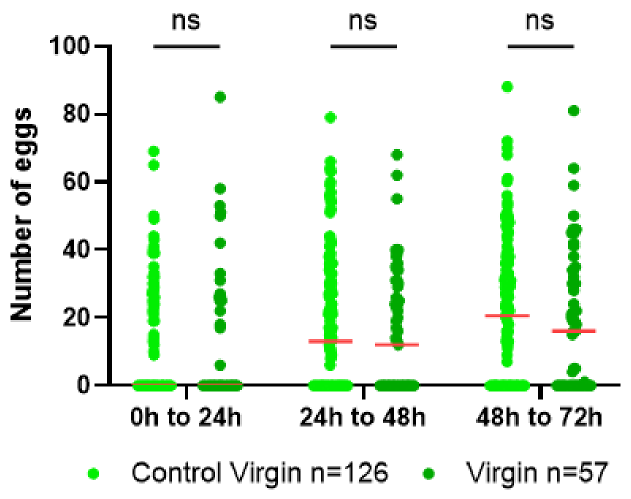

Supplementary Figure 1: Male presentation does not impact egg laying in virgin D. hydei females

Daily egg laying data were analyzed using a Mixed-effect model, for each day, conditions were compared using Šídák's multiple comparisons test. ns (P > 0.05)

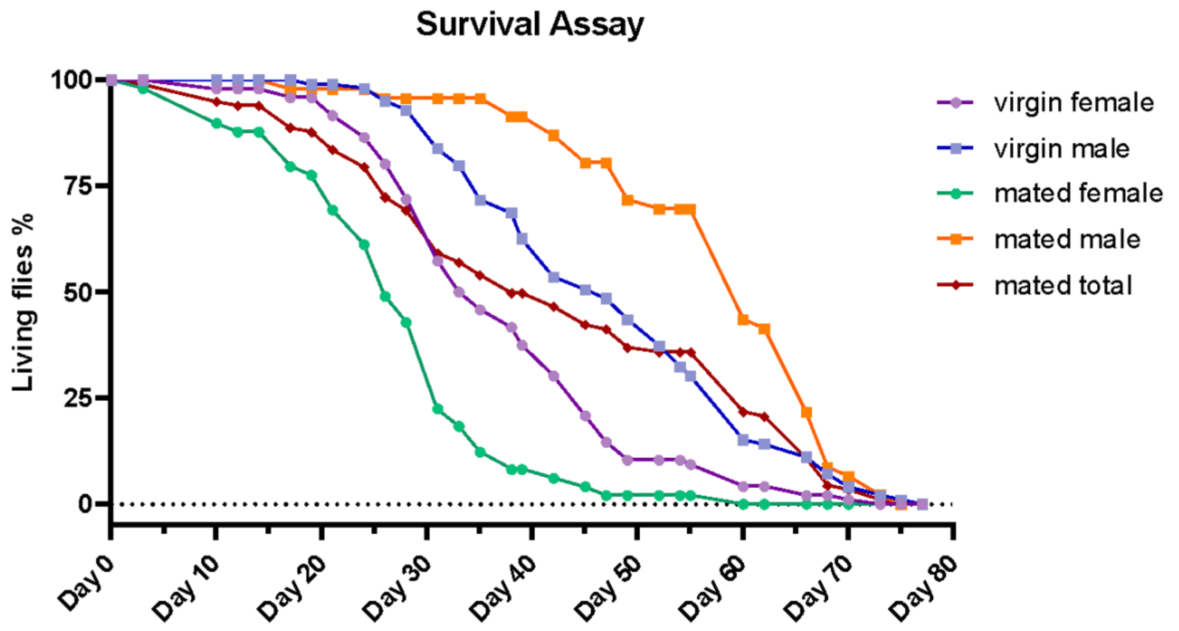

Supplementary Figure 2: Survival assay where mated females and mated males were housed together.

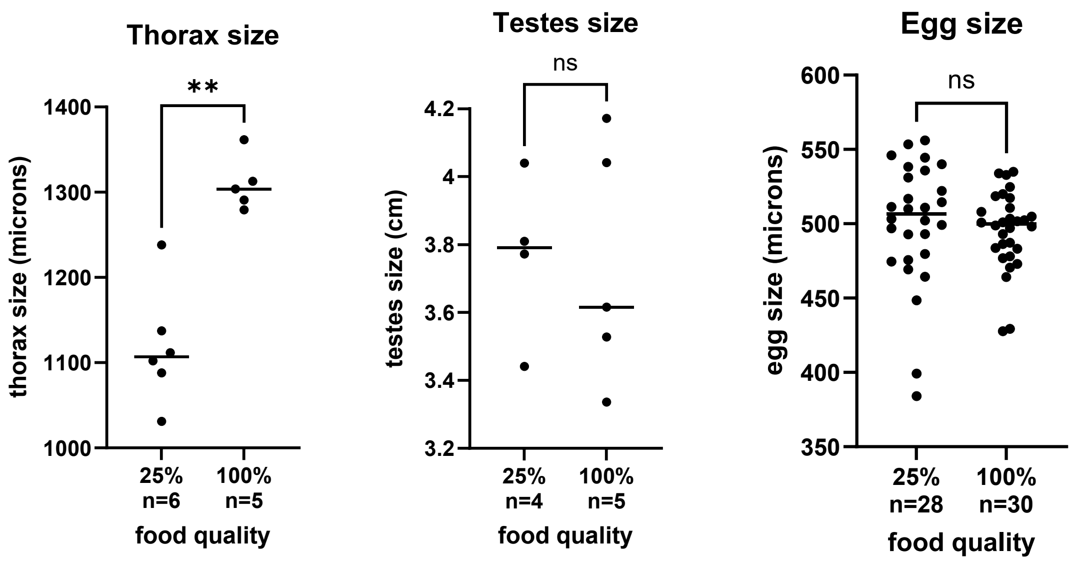

Supplementary Figure 3: Characteristics of flies after growth on lower quality food. Mann-Whitney tests were performed on each characteristic measured. Only thorax size was significantly affected by these manipulations.

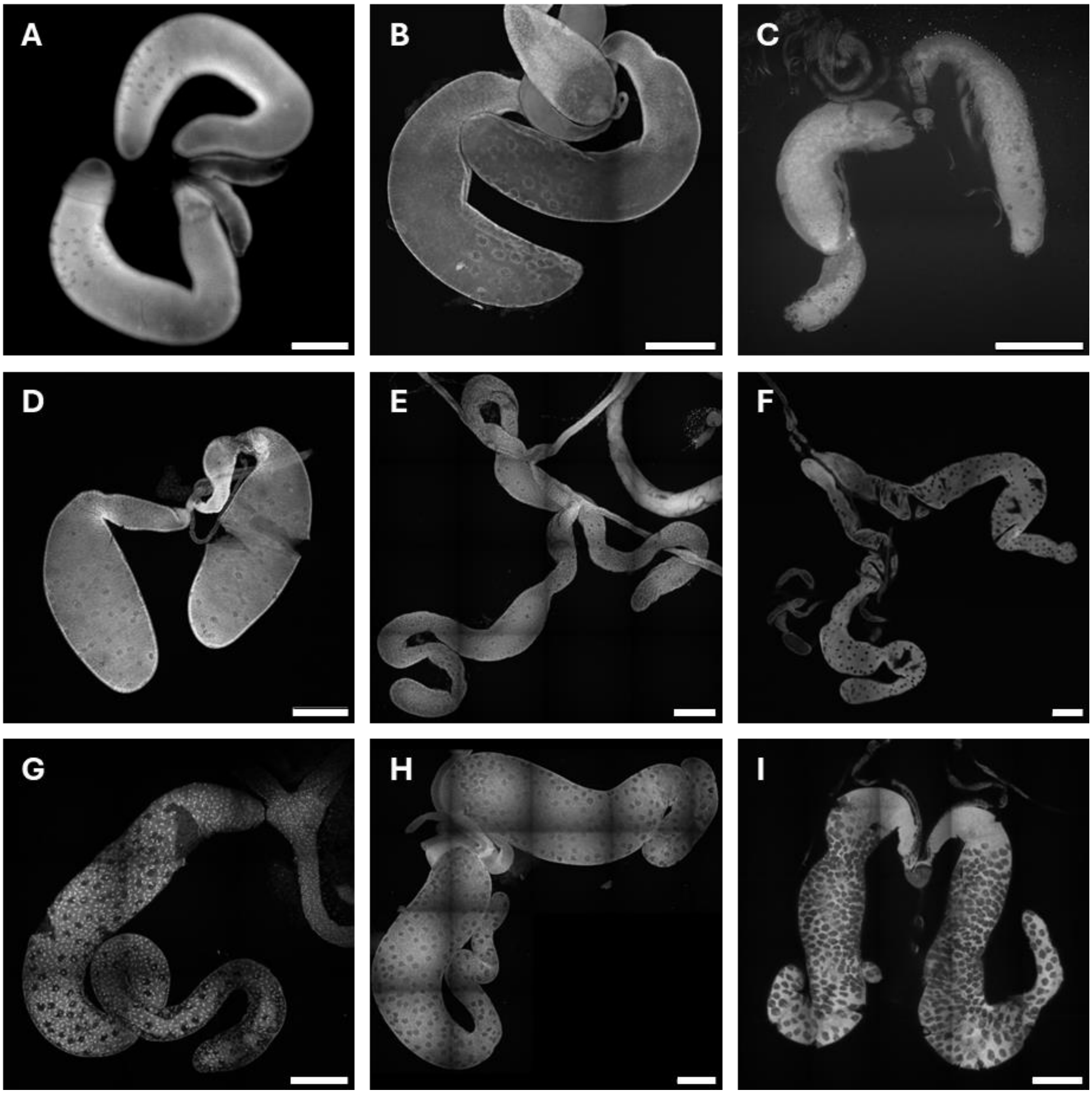

<u>Supplementary Figure 4: Accessory Glands of several Drosophila species</u>. A – *Scaptodrosphila lebanonensis*; B – *Drosophila melanogaster*; C – *Drosophila greeni*; D – *Drosophila ananassae*; E – *Drosophila virilis*; F – *Zaprionus indianus*; G – *Drosophila bifurca*; H – *Drosophila hydei*; I – *Drosophila nannoptera*. Ethidium Bromide staining, scale bars represent 200µm.

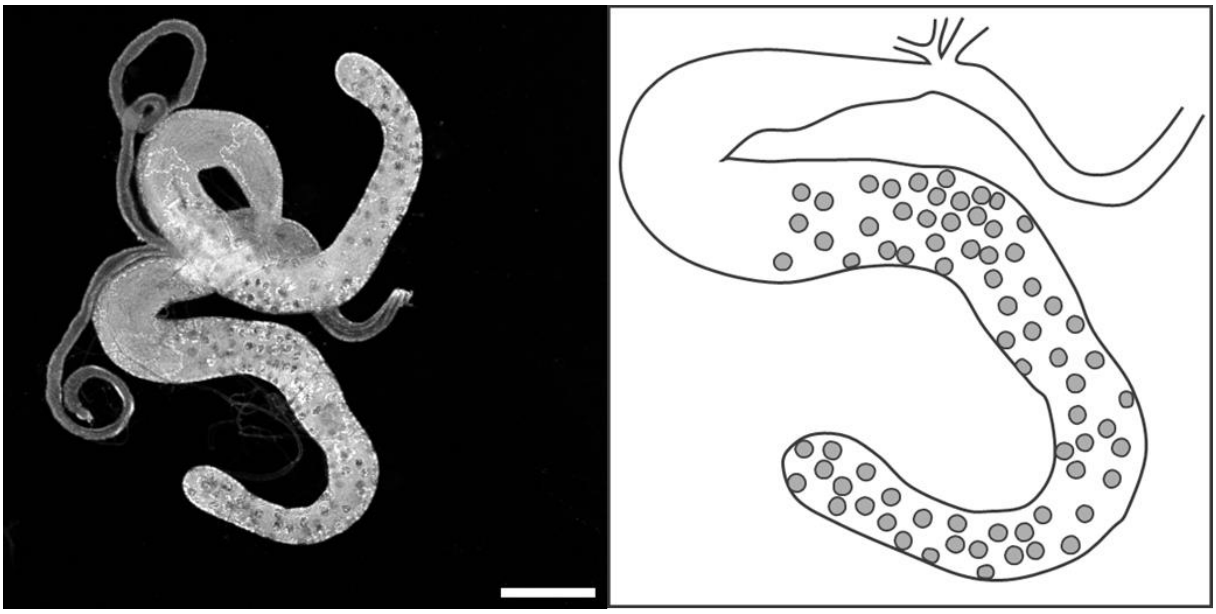

<u>Supplementary Figure 5: Accessory glands of Chymomyza pararufithorax</u>. Left: Ethidium Bromide staining, scale bars represent 200µm. Right: Representation of a gland lobe, with the secondary cells spread along most of the lobe length.

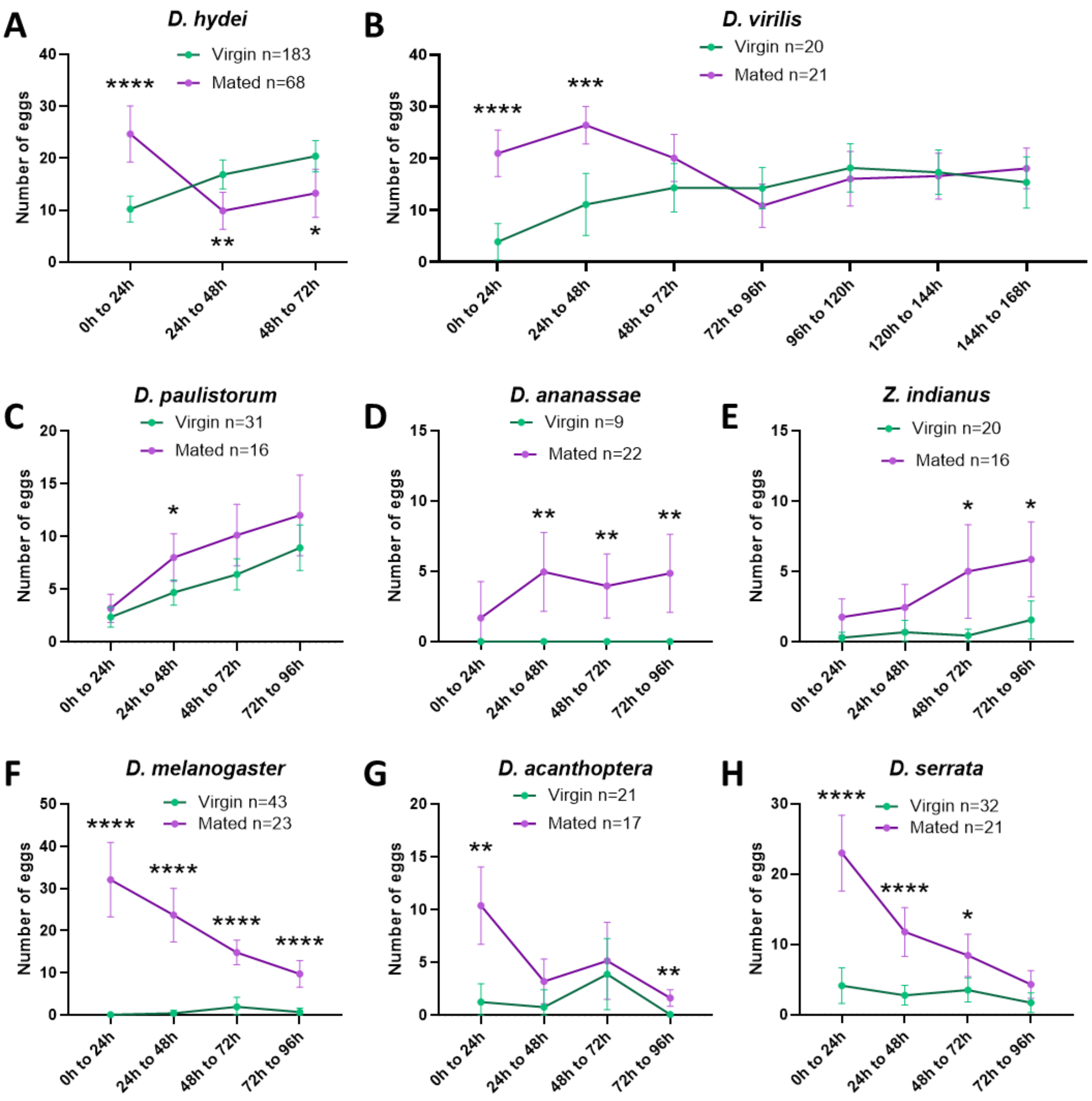

<u>Supplementary Figure 6: Analysis of the egg laying patterns of 8 Drosophila species in response to mating.</u> Daily egg laying data were analyzed using ANOVA2 or a Mixed-effect model, for each day, conditions were compared using Šídák's multiple comparisons test. ns (P > 0.05), * (P ≤ 0.01** (P ≤ 0.01), *** (P ≤ 0.001), **** (P ≤ 0.0001).

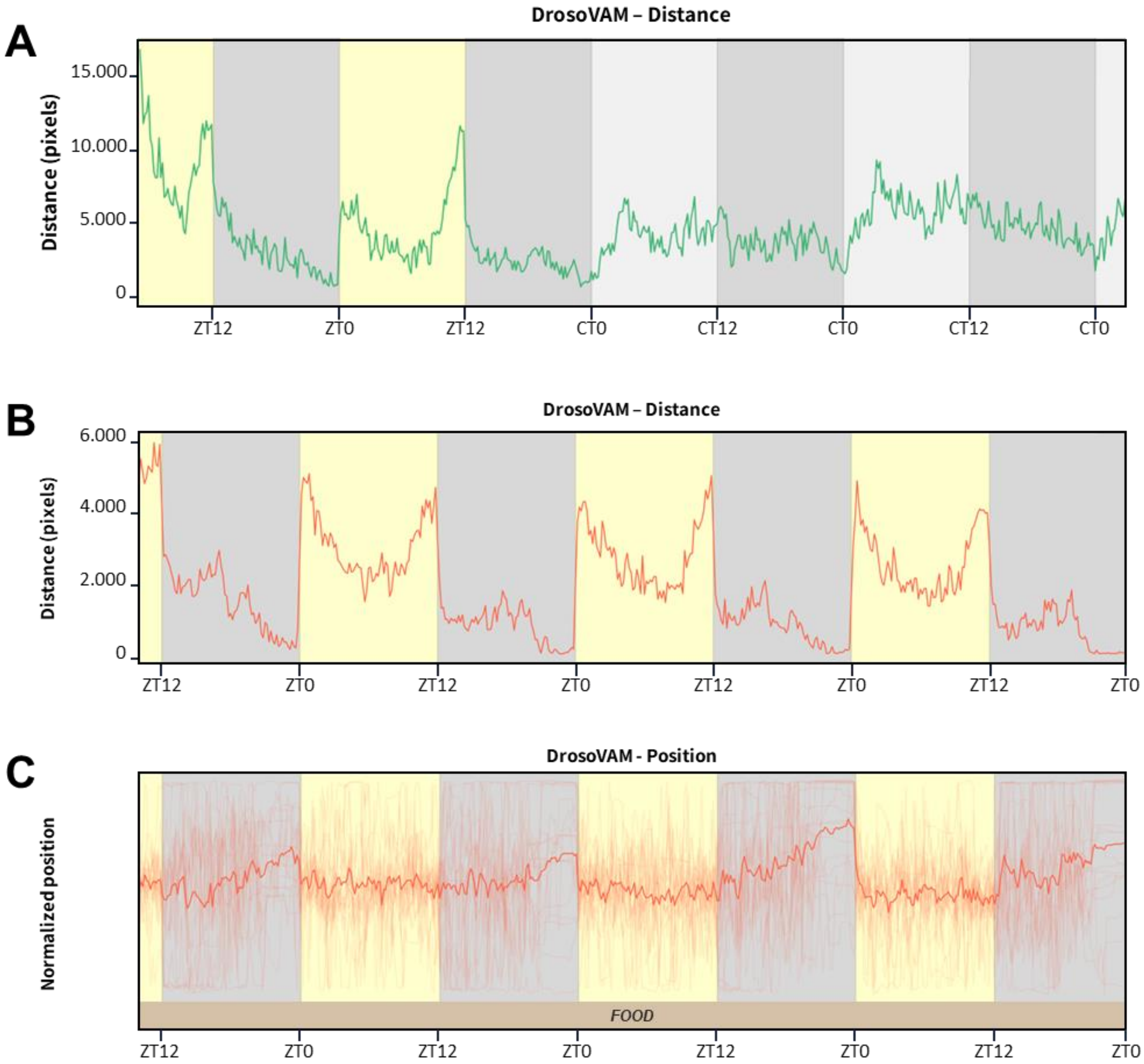

__Supplementary Figure 7: Further analysis of the circadian activity of D. hydei__. A. Flies were trained in 12h:12h Light/Dark cycles and then switched to constant darkness. Light/Dark cycles are shown with yellow/grey background respectively. In constant darkness, subjective day/night periods are shown with light grey/ grey background. Distance in pixels moved by virgin females, bins of 10 minutes. Circadian variations in the position relative to food of males *D. hydei*. B. Distance in pixels moved by males. C. Temporal representation of the position of the flies in the locomotor activity chambers. The extremity of the chamber in which food is present is located at the bottom of the chart. Positions of all the flies are shown (transparent lines) and the average position is represented with the solid line.

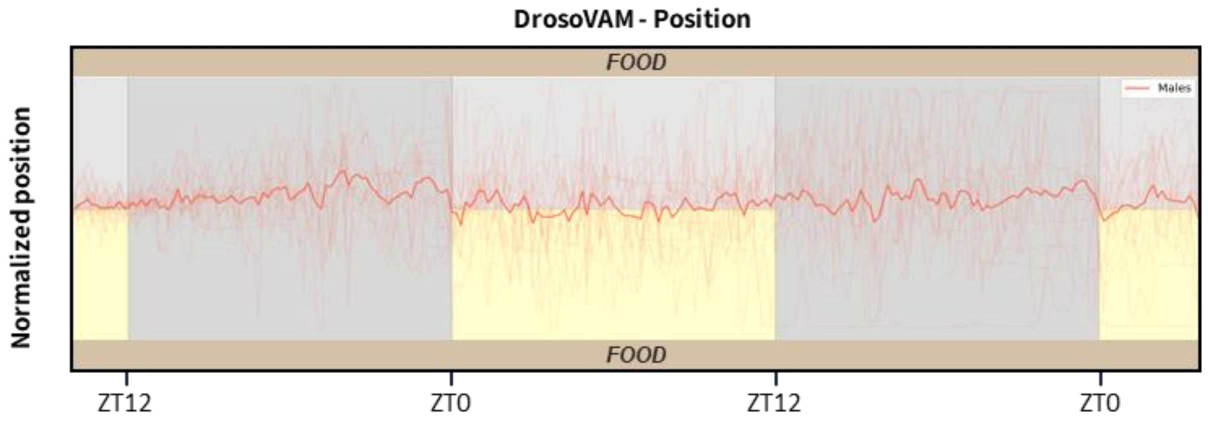

Supplementary Figure 8: Temporal representation of the position of the males in the darkness-preference assay chambers. Positions of all the flies are shown (transparent lines) and average position is represented with the solid line
