# Supplementary material for: Characterization of the post-mating responses of Drosophila hydei, a species that lacks Sex-Peptide
Source: Commun Biol. 2026 Apr 11;9:865. doi: 10.1038/s42003-026-10021-5 (PMC13315242; doi:10.1038/s42003-026-10021-5)
Supplement: Supplementary file 2 — Description of Additional Supplementary Files [file 42003_2026_10021_MOESM2_ESM.docx]

**Description of Additional Supplementary File**File name: Supplementary data 1
Description: All data used to generate the figures
