# Supplementary material for: Characterization of the post-mating responses of Drosophila hydei, a species that lacks Sex-Peptide
Source: Commun Biol. 2026 Apr 11;9:865. doi: 10.1038/s42003-026-10021-5 (PMC13315242; doi:10.1038/s42003-026-10021-5)
Supplement: Supplementary file 5 — Transparent Peer Review file [file 42003_2026_10021_MOESM5_ESM.pdf]

nature portfolio

Peer Review File

Characterization of the Post-Mating Responses of Drosophila hydei, a species that lacks Sex-Peptide

Corresponding Author: Dr Robert Maeda

This file contains all reviewer reports in order by version, followed by all author rebuttals in order by version.

Version 0:

Reviewer comments:

Reviewer #1

(Remarks to the Author)
In this study, the authors look at the postmating response of Drosophila hydei, one species out of many Drosophila that have lost the male accessory gland gene SP. They find that mating triggers the release of eggs that are already mature and ready to be laid within the first 24 hours, but there is no subsequent increase in egg-production, which is the long-term response induced by SP in D. melanogaster. They also find no differences in female postmating viability between virgin and mated females, which is different to the postmating response (PMR) detected by others in D. melanogaster. They also examined changes in circadian activity and sperm competition.

Overall, the work is nicely executed and has the strength of covering a wide variety of D. hydei PMR phenotypes. While the study offers a thorough analysis PMRs in D. hydei, it raised questions for me about its broader significance. There are many Drosophila species that have lost sex peptide, so I wonder whether the changes observed in PMR of D. hydei are more broadly shared with other species or specific to D. hydei. You should consider/cite work that has shown that D. melanogaster males lacking functional SP elicit a weak short-term response. Does this mean that species of the Drosophila genus that lack SP, not just D. hydei, should show PMRs similar to D. hydei? What about other genes that also influence PMRs (see minor comments -line 18)?

In my opinion, the authors examination of the length of the accessory gland lobes, and the number and position of secondary cells within the lobes, has a broader appeal because it includes sampling of other species of Drosophila instead of focusing solely on D. hydei. This analysis shows that the ancestral state of the gland might have been longer, with variation in the number and position of secondary cells (SCs) within the lobe. Interestingly, D. virilis which is a non-sophophora species with long AG lobes and large number of SCs has SP (Hopkins et al. 2024). Does D. virilis exhibit a PMR similar to D. melanogaster? If not, perhaps the long-term response in egg-laying is not just the consequence of having or not having SP but dependent on several proteins, like those that make up the SP-network or even others. In summary, the examination of so many PMRs in D. hydei offers the strength of a thorough description of PMRs in that species but leaves me wondering about the broader appeal. Enhancing the appeal of the work might involve leveraging phylogenetic patterns in morphology of the lobes of the glands, number and positioning of secondary cells, to strategically select Drosophila species for follow up PMR examination. This could focus on the most relevant or more commonly studied PMRs associated with SP, like perhaps the egg-laying response.

A few minor comments:
Line 18: Is it really true that PMRs rely on a single protein? What about ovulin? Or Acp36DE?

Line 45: "how many" instead of “how any”

Lines 54-57: In species outside the Drosophila genus, some of the PMRs might be independent of SP. Which begs the question of whether something similar might be true in many species of Drosophila.

Lines 163-164: Regarding your finding that D. hydei shows first male sperm precedence. The book “Sperm Competition” by Leigh Simmons has a table (2.3) that lists P2 values for many species. It shows that D. hydei is a sperm mixer with mean P2 = 0.5, ranging from 0.25-1.00. The P2 value is sensitive to time between successive matings. In other words, previous studies have already shown that D. hydeii does not show second male sperm precedence, and that the species is more of a

“mixer”. Two papers by Markow are cited (1985 Anim Behav and 1988 Func Ecol).

Lines 386-386: You seem to infer that SP duplication leads to relative more importance of the gene. However, a duplication can led to duplicates without functon(pseudogenes) or completely different functon(neofunctionalization). They might even lead to less importance of SP, if there is sub functionalization.

Line 448: “linked to” change to “associated with”

General comment: The discussion reads in many parts as repetition of results. I feel it would be more effective if you could minimize repetition of results.

Reviewer #2

(Remarks to the Author)
Revel et al.’s manuscript provides an important characterization of the post-mating response in Drosophlia hydei, a species of fly that is notable for at least two reasons. First, this species has remarkably long sperm (even by fly standards), making it an interesting potential species for studies of sexual selection and sperm-female coevolution. Second, as the authors discuss, it is a representative of the fly genus that lacks the sex peptide (SP) protein. Since this protein plays such an important role in the post-mating responses of the traditional model species D. melanogaster, it is an open and interesting question how PMRs vary in species that lack SP. For both of these reasons, then, the authors’ investigation of PMRs in D. hydei is timely and should be of interest to fly reproduction researchers.

Overall, the study is sound, thorough, and clearly described. I appreciate the mix of traditional fertility and behavioral assays and assays enabled by the advanced technology available to the authors. The primary suggestion I have for revision is to re-consider the framing of the manuscript. The introduction focuses on the idea that both D. melanogaster and D. hydei are cosmopolitan species and, thus, might experience similar selective pressures when it comes to some aspects of reproduction. While this might be true, it seems difficult to know how much each species’ reproduction would have been shaped by this recent similarly in environment versus the tens of millions of years of divergence that preceded it. Focusing on the cosmopolitan nature of D. hydei has other issues, as well, such as the fact that various “cosmopolitan” settings might nonetheless differ in habitats, seasonal temperatures, and other such variables. I think a stronger motivation would be to instead play up the large sperm and absence of SP, and to use these attributes to argue for D. hydei becoming an emerging model system for the comparative study of fly PMRs.

I offer some specific suggestions for revision below:

-Abstract line 20 and below: It’s not clear why fly species “living in similar environments” as D. melanogaster would be expected to have the same SP gene. The degree of phylogenetic relatedness is a more appropriate predictor. Additionally, since D. melanogaster is now common in many environments, living in similar environment seems somewhat meaningless. The abstract should be reframed away from “similar environments.” A similar issue occurs at line 63, where the authors note that both melanogaster and hydei are cosmopolitan, and this similar environment might exert similar pressures on their respective PMRs. This only holds to the extent to which their reproductive modes have shaped by their cosmopolitan-ness. Since the two species could only have been “cosmopolitan” for a very small fraction of their total divergence time, it isn’t clear how much their mating behaviors/responses would have been shaped by that limited amount of time, versus the previous tens of millions of years of divergence in other environments.

-Line 24: suggest adding “females” after the second instance of D. hydei, and clarifying “D. hydei females” or “females of this species” in line 26

-Lines 28-29: “choices that species can make” should be rephrased in terms of evolutionary selective pressures that have shaped different species’ reproductive behaviors/physiology.

-Line 36: It might be helpful to cite examples of other species where male SFPs are linked to PMR, before going into melanogaster specifically.

-Line 45: I’m not certain about framing the question of how PMRs work in non-SP containing species as whether they are “similar to those of melanogaster” is quite right. To what extent do we know that PMRs across all species that have SP are melanogaster-like? Unless all show mel-like PMR, then it’s not clear why the mel PMR specifically should be the thing against which non-SP-containing species should be compared. It might be useful to instead reframe this manuscript as a study that helps delineate the range of PMR in genus Drosophila, with D. hydei being an especially interesting species to study because of its lack of SP and its other unusual reproductive characteristics, such as its giant sperm. Both of those traits make the work compelling and interesting!

-Line 51: consider editing to “when males *or sperm* are not available” (given the prevalence of sperm storage in insects)

-Line 60: The comparison between hydei and mel here is useful; the authors could also add what is known about PMR/Sfps in other repleta group species.

-Wording suggestions for Fig. 1. Caption. At line 118: “females that have mated a single time, or more than once in rapid

succession" makes it sound like the comparison shown is between females mated 1 time vs. >1 time, but really the comparison in the graph is between virgins and females who mated 1 or more times in 2 hrs. Throughout, perhaps avoid referring to eggs laid by virgins "in the three days post-mating." Could rephrase to "eggs laid by equivalently aged virgin females over the same period" or some such.

-Line 154-5: rephrase to "why multiple mating has been selectively beneficial in D. hydei".

-Fig. 2B: The authors might also consider testing for whether the percentage of progeny from the first male is significantly greater than 50%. It probably is, as it looks like all trials came out that way, but a formal test of this might be useful for more definitively establishing first-male-precedence (under certain food conditions).

-Line 186: consider editing to "affects male size and male fertility".

-Fig. 2B: It might also be interesting to run the other control experiment, i.e., to ask if first-male precedence holds or goes away when both the first and the second male are reared in 25% nutrient food. I don't think this is essential, though.

-Line 265-6: consider rewording; it might be better to say that the SCs' function is to produce certain proteins (some of which relate to SP binding to sperm), rather than to say its role is directly in "SP attachment." Indeed, a common feature of the SCs across SP-containing and SP-lacking species could be producing a different subset of AG proteins than the main cells do.

-Lines 366-7: there is a redundant use of visible light blocking/almost infrared in this sentence.

-Line 435: Since the authors did not directly measure sperm storage, consider rephrasing this sentence. I think the safer conclusion is that male starvation did not affect the number of progeny produced, so to the extent that progeny production in their assay is indicative of sperm storage, storage was not affected. (Of course, if it is known whether D. hydei females store sperm in excess of the number of progeny they can produce in the time course of this assay, that information could be incorporated.)

-I like the discussion of alternative male strategies for maximizing reproductive success at the end of the discussion (lines 497-505). I wonder whether this information might be useful if the authors re-work the introduction as I describe in my comments above, such that the initial focus of the paper is more on the diversity of PMRs/mate guarding (chemical or physical) in flies, and less about the shared cosmopolitan ecology between mel and hydei, which is not discussed much in the discussion section.

-The methods section (lines 550-551) notes that the same (apparently single) male was used for female remating in the fertility assays described in Fig. 1. Since this assay found no significant difference between females mated singly vs. multiply, I am wondering whether a potential explanation could be sperm exhaustion of the single male. Would the results be different if the females remated with fresh males instead? Or is there literature on D. hydei sperm exhaustion that could negate this issue? I would recommend the authors address this experimental design choice explicitly in the relevant part of the results section (lines 125-128).

-Fig. S2: What is a "non-parametric t-test"? I think t-tests are parametric by definition, so please state the name of the actual non-parametric test used (e.g., Mann-Whitney U?).

Reviewer #3

(Remarks to the Author)
This manuscript is a valuable study that fills a gap in the literature regarding the post-mating responses of Drosophila hydei. This is a species which lack in Sex Peptide, a protein that induces the most known post-mating responses in many of the Sophophora group species. There's a lot of interesting data in this paper and it's well worth publishing. The data will be very useful for researchers in the field going forward and it is a rich dataset. Overall, the paper is pretty well written and easy to read, but there are a number of ways in which it could potentially be improved.

General comments:

• The abstract seems to focus on a rather small subsection if the results. It's a shame more of the nice findings aren't articulated here (or hinted at in the title); it risks that the paper will get often overlooked in literature searches when it actually contains lots of things that would interest a wide audience.
• The paper could be much more succinct. The results section is extremely wordy and encompasses a lot of discussion. As such when we get to the discussion, there is a lot of repetition making an overall length of the paper much longer than it really needs to be. A combined results and discussion section would be better if the journal style allows it.
• Some of the thinking around the actions of the ejaculate in D. melanogaster – at least from a neurogenetics perspective – is that the default female state is the mated state; virgins suppress egg laying and refractoriness, and SP releases that suppression. So one potential viewpoint would be that D. hydei partially lacks that virgin suppression state. This may be worth some discussion.
• Overall, there's a strong effort emphasis on the differences between species, but there are also quite a lot of similarity between the species. The PMR similarities might be regarded as quite surprising, given the very different mating system and sperm length. The paper would, IMO, be better balanced if it recognised both the similarities as well as the differences.
• The statement that "D. hydei do not increase egg production" seems a little misleading. Very clearly mating does lead to an

increase in egg production, it's just that it's over a short period of time, and followed by a decrease relative to virgins. In some ways that it's not so dissimilar to D. melanogaster, where at least in some strains virgin females will begin laying eggs at quite a high rate as they get older (there is genetic variability in this: some populations retaining eggs as virgins more strongly than others). At some point virgin egg laying overtakes that of mated females (e.g. day 16 in one of the experiments in this paper https://doi.org/10.1016/0022-1910(86)90140-X ; there are lots of other 20th century papers that look at the same thing). So, it is certainly on a very different time scale in the different species – but it could be argued that it's not entirely distinct phenomenon.
• The fact that multiple mating does not further increase egg laying (Fig 1C) is also arguably consistent with, not contradictory to, D. melanogaster.
• "….consistent with the idea that mating does not trigger an increase in oogenesis but instead triggers a short-term egg laying response to release stored eggs". Don't D. melanogaster also do this as part of the PMR? The ovulin response? So again, in some respects it seems that the strategy is not so different between the species.
• Sample sizes seem to be given for some experiments but not others – make Ns clear for all the experiments
• Some rather sloppy language and grammar throughout (see minor comments). In particular when naming Drosophila melanogaster and Drosophila hydei, sometimes it is written as D. melanogaster/hydei and sometimes only like melanogaster/hydei. Please, write it as Drosophila melanogaster or Drosophila hydei the first time and D. melanogaster or D. hydei on the following ones.
• Why is sample size of virgins 3x more than mated in Fig 1b? Could you please clarify this difference in the main text?
• Figure 2B. Is a t-test the best way of analysing P1 (paternity sired by the first male)?
• In the sperm competition experiment (see discussion Line 438-443) it is discussed that sperm from starved males is less competitive than that of unstarved ones, based solely on the progeny sired by the female. If sperm count has not been possible to measure it is difficult to disentangle if the response was mediated by postcopulatory competition or female choosiness. Please, further discuss this.
•

Minor:
• gene names should be italicised
• final sentence of the abstract is rather vague "These changes impacting characteristics like lifespan and sperm selection demonstrate the variety of choices that species can make and be successful in a given environment". Not sure what point this is supposed to make.
• L33-34: this phrasing sounds rather group-selectionist: selection acts on individuals > groups > species.
• L39. This is first mention of seminal fluid proteins in main text, so introduce the acronym SFPs here (instead of L39)
• L39 onwards: use "D." instead of Drosophila when talking about species.
• L43. Here you're talking about the SP gene: make this clear and italicise SP
• L45 onwards: make sure "D." comes in front of melanogaster or hydei
• L52 "debatably"? I think one would have to be quite contrarian to argue that female refractoriness was not advantageous to the previous male
• L71 "Perhaps due to this selection for long sperm, a second significant difference from melanogaster is the loss of the SP gene". I don't follow the logic of how selection on sperm length should lead to the loss of SP
• L79 D. hydei
• L92 "yet equally successful". What on is that supposed to mean? How do you rate the success of two different species?
• L105. Specify if the second female you are comparing them to is virgin or not, as it might be confusing for the reader.
• L201. Fewer, not less.
• L214 "IV – The absence of SP has no direct consequences on the Male Accessory Gland shape or structure". This seems somewhat of an overinterpretation. Given that no direct manipulation of SP has been performed, I would change this title, or at least talk circumspection about associations.
• L219 D.
• L258. True but SP is not made in the SCs.
• L303 D.
• L340, In D. melanogaster, SP
• L343-345. This sentence is incomplete and doesn't make sense.
• L379 D.
• Line 380. Drosophilids -> drosophilids
• L383 SP
• Line 384. "Drosophila" should be in italics
• Line 388. "Drosophila" should be in italics
• L389 D.
• Line 440. "melanogaster" should go in italics
• Line 451. "melanogaster" should be in italics
• L484 Whys suddenly Secondary Cells and not SCs?
• L492/3 sperm not sperms
• L506. "The differences of the PMR observed in D. hydei compared to what is known in D. melanogaster suggests that many reproductive strategies may be equally valid in the wild". Seems a rather meaningless statement. What does "valid" mean here?

Reviewer #4

(Remarks to the Author)

I co-reviewed this manuscript with one of the reviewers who provided the listed reports. This is part of the Communications Biology initiative to facilitate training in peer review and to provide appropriate recognition for Early Career Researchers who co-review manuscripts.

Version 1:

Reviewer comments:

Reviewer #2

(Remarks to the Author)
I appreciate the authors' careful attention to all of the reviewer comments. My main suggestion was to reconsider how the study was framed, and I think the new version is nicely improved in this area. I have just a few small suggestions, but feel the manuscript is now in good shape for publication. (I do not feel like I need to see the manuscript again.) Thanks to the authors for this interesting study, which I continue to feel will be an important one for the field.

Line 55-59: I really like the framing of the key questions here – thanks for this good revision! I also like the description (lines 73-91) of the interesting, relevant facets of D. hydei reproductive biology.

Line 287 and Fig. 4: The text says that AG lobe length was "no more than 1000 um" for the Sophophora group, but in Fig. 4B, it seems that a good number of the data points in the yellow-shaded region have lengths between 1000-2000 um. Please double check.

Line 511-513: In addition to changes in the location and copy number of the SP gene in the melanogaster group, I believe a finding of the Tsuda et al. paper (ref 16) was that the SP receptor (SPR) also became much more highly expressed in the female reproductive tract within the melanogaster group. This might also be a contributing factor to why SP plays a more central role in mel-group species. Perhaps this is also worth a brief mention at this point in the discussion?

Small editing suggestions:
Line 41: consider changing "have been found" to "have been identified", since "found" was used previously in the sentence
Line 44: check for consistency across the manuscript in how male accessory gland is abbreviated (mAG vs. MAG).
Line 131-133: Consider inverting the order to better match the experimental result: this means D. hydei females will usually have a stock of eggs to lay upon mating (consistent with the observed uptick in laying in the first 24 hrs), but might occasionally have only a few available
Line 171-2: consider rewording "males or the act of constant mating" to "constant exposure to males or more frequent mating"
Line 205: "sperm seems" --> "sperm seem"
Line 358-9: check sentence fragment at the end

Reviewer #4

(Remarks to the Author)
I believe the authors have enhance the manuscript by implementing the suggestions made by the reviewers. I, therefore, think this paper is worth of publishing. Please see some minor comment bellow.

-The abstract has been rewritten showing a wider understanding of the results found in this study, which will potentially catch the attention of more readers.
-The length of the paper is still quite long, however we believe that the amount of data gathered in the study is a lot and needs to be explained. I thank the authors for joining the results and discussion sections making it less repetitive. Unless the editorial have any concerns with the length, we think is has been improved.
-There is more balance now throughout the ms between differences and similarities in both species.
-Language is much better now.
-Some of the general comments and minor comments have been addressed.

-Previous comment: "Some of the thinking around the actions of the ejaculate in D. melanogaster – at least from a neurogenetics perspective – is that the default female state is the mated state; virgins suppress egg laying and refractoriness, and SP releases that suppression. So one potential viewpoint would be that D. hydei partially lacks that virgin suppression state. This may be worth some discussion."

I can't see this has been explained somehow and it could be a very interesting point to further develop.

-Previous comment: "The statement that "D. hydei do not increase egg production" seems a little misleading. Very clearly mating does lead to an increase in egg production, it's just that it's over a short period of time, and followed by a decrease relative to virgins. In some ways that it's not so dissimilar to D. melanogaster, where at least in some strains virgin females will begin laying eggs at quite a high rate as they get older (there is genetic variability in this: some populations retaining eggs as virgins more strongly than others). At some point virgin egg laying overtakes that of mated females (e.g. day 16 in one of the experiments in this paper https://doi.org/10.1016/0022-1910(86)90140-X ; there are lots of other 20th century papers that look at the same thing). So, it is certainly on a very different time scale in the different species – but it could be argued that it's not entirely distinct phenomenon."

-The title of the first result “Mated females show a short-term egg laying burst with no increase in egg production” is still misleading, however later the text they argue that it is a short-term effect and there are similarities between D. hydei and D. melanogaster.

-Previous comment: “Why is sample size of virgins 3x more than mated in Fig 1b? Could you please clarify this difference in the main text?”

I can’t see this in the new ms

-Previous comment: “In the sperm competition experiment (see discussion Line 438-443) it is discussed that sperm from starved males is less competitive than that of unstarved ones, based solely on the progeny sired by the female. If sperm count has not been possible to measure it is difficult to disentangle if the response was mediated by postcopulatory competition or female choosiness. Please, further discuss this.“

I don’t think this has been done, I would be more circumspect about this assumption.

New minor comments:
• 54. Change ‘Drosophilids’ to ‘drosophilids’
• Line 230-240. Some this sentence has been included in the title IV, I assumed by mistake.
• Line 487-489. The title ‘V-Outlook on the reproductive strategy of D. hydei’ has different fonts

Open Access This Peer Review File is licensed under a Creative Commons Attribution 4.0 International License, which permits use, sharing, adaptation, distribution and reproduction in any medium or format, as long as you give appropriate credit to the original author(s) and the source, provide a link to the Creative Commons license, and indicate if changes were made.
In cases where reviewers are anonymous, credit should be given to 'Anonymous Referee' and the source.
The images or other third party material in this Peer Review File are included in the article’s Creative Commons license, unless indicated otherwise in a credit line to the material. If material is not included in the article’s Creative Commons license and your intended use is not permitted by statutory regulation or exceeds the permitted use, you will need to obtain permission directly from the copyright holder.
To view a copy of this license, visit https://creativecommons.org/licenses/by/4.0/

Dear Editors of Communications Biology,

We thank you and the reviewers for the time and effort placed in the review our manuscript entitled "Characterization of the Post-Mating Responses of *Drosophila hydei*, a species that lacks Sex-Peptide" by Revel et al. (COMMSBIO-25-8192-T). Overall, we were very happy that the reviewers valued our work and made very useful suggestions to help us improve our manuscript. We have tried our best to incorporate these suggestions. Primary to these changes are a substantial reorganization of the manuscript and the addition of experiments detailing the egg-laying post-mating response in other Drosophila species. Please find below a detailed response to each of the reviewer points.

Reviewer #1 (Remarks to the Author):

I*n this study, the authors look at the postmating response of Drosophila hydei, one species out of many Drosophila that have lost the male accessory gland gene SP. They find that mating triggers the release of eggs that are already mature and ready to be laid within the first 24 hours, but there is no subsequent increase in egg-production, which is the long-term response induced by SP in D. melanogaster. They also find no differences in female postmating viability between virgin and mated females, which is different to the postmating response (PMR) detected by others in D. melanogaster. They also examined changes in circadian activity and sperm competition.*

Overall, the work is nicely executed and has the strength of covering a wide variety of D. hydei PMR phenotypes. While the study offers a thorough analysis PMRs in D. hydei, it raised questions for me about its broader significance. There are many Drosophila species that have lost sex peptide, so I wonder whether the changes observed in PMR of D. hydei are more broadly shared with other species or specific to D. hydei. You should consider/cite work that has shown that D. melanogaster males lacking functional SP elicit a weak short-term response. Does this mean that species of the Drosophila genus that lack SP, not just D. hydei, should show PMRs similar to D. hydei? What about other genes that also influence PMRs (see minor comments -line 18)?

<u>Response</u>: We thank this reviewer for this question and their kind comments. Indeed, we came to this work almost assuming that *D. hydei* would display many of PMRs found in *D. melanogaster* but maybe use different molecules to establish them. This is, of course not the case. To address the broader significance, we have rewritten much of the manuscript, focusing more on the long-sperm characteristic of *D. hydei* (as suggested by the reviews). In relation to the final aspect of this set of questions, regarding if other species without SP would behave similarly to *D. hydei*, we believe that there will probably be many strategies that can successfully be selected to increase reproductive success. Perhaps, *hydei* and other long sperm species may have evolve them as a way for females to select better males. This change seems to have had consequences on many aspects of the *D. hydei* life, including the PMRs that we have described. In support of the various changes in PMRs, we have also included a new supplementary figure, where we present our data in examining the egg-laying PMRs of six additional species. Five of the species examined still have the *SP* gene (in different genomic locations), while one species does not have *SP*. While we could not fully

examine the PMRs in these species due to practical constraints, these experiments do show that there are many different egg-laying responses to mating. To explain our feelings on this issue, we have added as section to the end of the manuscript discussing this point.

In my opinion, the authors examination of the length of the accessory gland lobes, and the number and position of secondary cells within the lobes, has a broader appeal because it includes sampling of other species of Drosophila instead of focusing solely on D. hydei. This analysis shows that the ancestral state of the gland might have been longer, with variation in the number and position of secondary cells (SCs) within the lobe. Interestingly, D. virilis which is a non-sophophora species with long AG lobes and large number of SCs has SP (Hopkins et al. 2024). Does D. virilis exhibit a PMR similar to D. melanogaster? If not, perhaps the long-term response in egg-laying is not just the consequence of having or not having SP but dependent on several proteins, like those that make up the SP-network or even others. In summary, the examination of so many PMRs in D. hydei offers the strength of a thorough description of PMRs in that species but leaves me wondering about the broader appeal. Enhancing the appeal of the work might involve leveraging phylogenetic patterns in morphology of the lobes of the glands, number and positioning of secondary cells, to strategically select Drosophila species for follow up PMR examination. This could focus on the most relevant or more commonly studied PMRs associated with SP, like perhaps the egg-laying response.

Response:
The seed of this project was to try to do exactly what you suggest here. I guess great minds… This is why we have the egg-laying data mentioned above, which came from our initial experiments. Our initial goal was to correlate the evolution of *SP*, the *SP*-network proteins, mAG structure and PMRs. Unfortunately, this proved to be difficult, and we were also somewhat scooped by Hopkins 2024, regarding *SP* conservation. Some of the other problems we faced were the species we could obtain (about 40) and how to grow each of these species in our lab. Next, we found many differences in each organism both gross and subtle. Knowing when to perform assays, how old each individual should be, what food to use, and establishing general baselines, soon became a monumental task for our small lab of two full-time scientists. Then, it soon became obvious that we could not only look for melanogaster-like behaviors. Not only were they less apparent than we originally expected (outside of the *melanogaster* group), but how we design tests for each aspect often required a complicated decision matrix. We ended up concluding that we would be better off focusing on one organism and developing assays that might "catch" details that might otherwise be missed.
Still, as mentioned above, we have included new egg laying data from other species to help address this comment and have modified the text to include some discussion of this topic.

A few minor comments:
Line 18: Is it really true that PMRs rely on a single protein? What about ovulin? Or Acp36DE?

Response: We never meant to say that all PMRs rely on one protein, but that most of the studied PMRs in *D. melanogaster* are impacted by SP. This has been corrected, and we do

discuss *ovulin* and *Acp36DE*.

Line 45: "how many" instead of "how any"
Response: This has been corrected

Lines 54-57: In species outside the Drosophila genus, some of the PMRs might be independent of SP. Which begs the question of whether something similar might be true in many species of Drosophila.
Response: In fact, this must be true, as *SP* is a relatively recent addition. In fact, that is part of our logic that there should be PMRs not linked to SP. It is only that they might be hidden, and/or changed, in *D. melanogaster* as the SP mechanisms are so strong. We tried to make this clearer in the new text.

Lines 163-164: Regarding your finding that D. hydei shows first male sperm precedence. The book "Sperm Competition" by Leigh Simmons has a table (2.3) that lists P2 values for many species. It shows that D. hydei is a sperm mixer with mean P2 = 0.5, ranging from 0.25-1.00. The P2 value is sensitive to time between successive matings. In other words, previous studies have already shown that D. hydei does not show second male sperm precedence, and that the species is more of a "mixer". Two papers by Markow are cited (1985 Anim Behav and 1988 Func Ecol).

Response: Thank you. We are always happy when our results are consistent with the literature. We have added the first of these references as the second talks about *D. mojavensis*.

Lines 386-386: You seem to infer that SP duplication leads to relative more importance of the gene. However, a duplication can led to duplicates without functon(pseudogenes) or completely different functon(neofunctionalization). They might even lead to less importance of SP, if there is sub functionalization.
Response: True, this may be the case. Hopkins 2024 was able to find expression data for 42 *SP* homologues, many of which were expressed in a sex specific fashion. There were a couple of cases where pseudogenization might be happening, but for the most part, most of the duplicates seem to be real genes. This does not mean they all function in the same fashion. However, it is also true that not all cases of gene duplication are simply to provide evolution with material to create change (for example, the histone genes and the *HSP70* genes to some extent, all mostly function in the same way). The fact that gene duplications were stably established would either mean that there is some increased error rate in replication at this area, or that it is somehow advantageous (given the frequency) to duplicate this gene. Whether this is subsequently used for an evolutionary neofunctionalization is beside the fact that the duplication did first occur.
With the *SP* genes, there is some evidence that they may be undergoing rapid modifications, like many SFPs. The fact that many of the known homologues show similar tissue expression specificity suggests a role for these proteins in reproduction. Hopkins et al. 2024 proposed that these duplications may be evidence of the sexual conflict taking place between males and females regarding *SP*. By making many versions of *SP*, the male can ensure an advantage, perhaps by creating effects via alternative receptors.

Thus, while we cannot definitively say that the increased copy number means increased importance, we feel the correlation between gene duplication events, the genomic change of location and the prominence of SP response in these species, as shown by Tsuda et al. do hint that this is the case. We discuss this a bit in the manuscript and hope that we do not stress this point too much.

Line 448: "linked to" change to "associated with"
Response: Changed

General comment: The discussion reads in many parts as repetition of results. I feel it would be more effective if you could minimize repetition of results.

Response: Sorry about this. One of the authors (RM) was always told that repetition in papers is good as people do not always read a paper all through at once. Given this comment, we have tried to remove some repetitive elements (but it is hard to break habits).

Reviewer #2 (Remarks to the Author):

Revel et al.'s manuscript provides an important characterization of the post-mating response in Drosophlia hydei, a species of fly that is notable for at least two reasons. First, this species has remarkably long sperm (even by fly standards), making it an interesting potential species for studies of sexual selection and sperm-female coevolution. Second, as the authors discuss, it is a representative of the fly genus that lacks the sex peptide (SP) protein. Since this protein plays such an important role in the post-mating responses of the traditional model species D. melanogaster, it is an open and interesting question how PMRs vary in species that lack SP. For both of these reasons, then, the authors' investigation of PMRs in D. hydei is timely and should be of interest to fly reproduction researchers.

Overall, the study is sound, thorough, and clearly described. I appreciate the mix of traditional fertility and behavioral assays and assays enabled by the advanced technology available to the authors. The primary suggestion I have for revision is to re-consider the framing of the manuscript. The introduction focuses on the idea that both D. melanogaster and D. hydei are cosmopolitan species and, thus, might experience similar selective pressures when it comes to some aspects of reproduction. While this might be true, it seems difficult to know how much each species' reproduction would have been shaped by this recent similarly in environment versus the tens of millions of years of divergence that preceded it. Focusing on the cosmopolitan nature of D. hydei has other issues, as well, such as the fact that various "cosmopolitan" settings might nonetheless differ in habitats, seasonal temperatures, and other such variables. I think a stronger motivation would be to instead play up the large sperm and absence of SP, and to use these attributes to argue for D. hydei becoming an emerging model system for the comparative study of fly PMRs.

Response:
We thank this reviewer for their insight and their kind review. We have rewritten/reorganized the manuscript to try accommodating for the major suggestions. We hope that this reviewer finds our manuscript improved by our changes.

-Abstract line 20 and below: It's not clear why fly species "living in similar environments" as D. melanogaster would be expected to have the same SP gene. The degree of phylogenetic relatedness is a more appropriate predictor. Additionally, since D. melanogaster is now common in many environments, living in similar environment seems somewhat meaningless. The abstract should be reframed away from "similar environments." A similar issue occurs at line 63, where the authors note that both melanogaster and hydei are cosmopolitan, and this similar environment might exert similar pressures on their respective PMRs. This only holds to the extent to which their reproductive modes have shaped by their cosmopolitan-ness. Since the two species could only have been "cosmopolitan" for a very small fraction of their total divergence time, it isn't clear how much their mating behaviors/responses would have been shaped by that limited amount of time, versus the previous tens of millions of years of divergence in other environments.

Response: We originally thought that environment could have a strong impact on evolution and changes regarding reproductive strategies, we thus looked for species that live in similar conditions (for example *D. grimshawi* is another species that may have a strikingly different

reproductive biology than *D. melanogaster*, but the two species do not share the same environmental niche, therefore making it a less compelling species for a comparative study…). As the reviewer found this less compelling, we have tried to limit these elements in the revised manuscript.

-Line 24: suggest adding "females" after the second instance of D. hydei, and clarifying "D. hydei females" or "females of this species" in line 26
Response: Changed

-Lines 28-29: "choices that species can make" should be rephrased in terms of evolutionary selective pressures that have shaped different species' reproductive behaviors/physiology.

Response: The abstract has been changed, and we tried to make this point clearer.

-Line 36: It might be helpful to cite examples of other species where male SFPs are linked to PMR, before going into melanogaster specifically.

Response: We added some talk elements regarding the role of SFPs beyond *Drosophila*.

-Line 45: I'm not certain about framing the question of how PMRs work in non-SP containing species as whether they are "similar to those of melanogaster" is quite right. To what extent do we know that PMRs across all species that have SP are melanogaster-like? Unless all show mel-like PMR, then it's not clear why the mel PMR specifically should be the thing against which non-SP-containing species should be compared. It might be useful to instead reframe this manuscript as a study that helps delineate the range of PMR in genus Drosophila, with D. hydei being an especially interesting species to study because of its lack of SP and its other unusual reproductive characteristics, such as its giant sperm. Both of those traits make the work compelling and interesting!

Response: Thank you for this comment. We completely agree about this fallacy and yet it is what we believed at the beginning of this work. This comes from the common statement that known PMRs are an attempt to "maximize" reproductive success. In our simple minds that led to a feeling that the responses in *D. melanogaster* approached maximal success for a species living in that ecosystem. In any case, we now know better. The framing of the paper is now oriented toward a more descriptive study of *D. hydei*, taking a comparative approach with *D. melanogaster* as it is the "reference" species when studying PMRs in fruit flies. We have also added substantial discussion of the phenotypes relative to the long-sperm characteristic of this species.

*-Line 51: consider editing to "when males *or sperm* are not available" (given the prevalence of sperm storage in insects)* –

Response: We have added this. Thank you.

-Line 60: The comparison between hydei and mel here is useful; the authors could also add what is known about PMR/Sfps in other repleta group species.

Response: We added a bit about this, though not much seems to be known.

-Wording suggestions for Fig. 1. Caption. At line 118: "females that have mated a single time, or more than once in rapid succession" makes it sound like the comparison shown is between females mated 1 time vs. >1 time, but really the comparison in the graph is between virgins and females who mated 1 or more times in 2 hrs. Throughout, perhaps avoid referring to eggs laid by virgins "in the three days post-mating." Could rephrase to "eggs laid by equivalently aged virgin females over the same period" or some such.
Response: Thank you. The figure caption was corrected.

-Line 154-5: rephrase to "why multiple mating has been selectively beneficial in D. hydei". -
Response: Thank you. This has been corrected

-Fig. 2B: The authors might also consider testing for whether the percentage of progeny from the first male is significantly greater than 50%. It probably is, as it looks like all trials came out that way, but a formal test of this might be useful for more definitively establishing first-male-precedence (under certain food conditions).

Response: We are not sure how to do this. If we perform a simple binomial test for scores greater than 50%, we definitely see significance to the 100%100% category and not the 25%:100%. We could include this if desired.

-Line 186: consider editing to "affects male size and male fertility".
Response: Thank you. The figure caption was corrected.

-Fig. 2B: It might also be interesting to run the other control experiment, i.e., to ask if first-male precedence holds or goes away when both the first and the second male are reared in 25% nutrient food. I don't think this is essential, though.

Response: While we would like to do this, unfortunately, financial support for this project has essentially pulled.

-Line 265-6: consider rewording; it might be better to say that the SCs' function is to produce certain proteins (some of which relate to SP binding to sperm), rather than to say its role is directly in "SP attachment." Indeed, a common feature of the SCs across SP-containing and SP-lacking species could be producing a different subset of AG proteins than the main cells do.
Response: Thank you. We tried to state this in a much more nuanced fashion in the new version

-Lines 366-7: there is a redundant use of visible light blocking/almost infrared in this sentence.
Response : Changed the sentence for improved clarity.

-Line 435: Since the authors did not directly measure sperm storage, consider rephrasing this sentence. I think the safer conclusion is that male starvation did not affect the number of

progeny produced, so to the extent that progeny production in their assay is indicative of sperm storage, storage was not affected. (Of course, if it is known whether D. hydei females store sperm in excess of the number of progeny they can produce in the time course of this assay, that information could be incorporated.) –

Response: We rephrased this to make the statement clearer.

-I like the discussion of alternative male strategies for maximizing reproductive success at the end of the discussion (lines 497-505). I wonder whether this information might be useful if the authors re-work the introduction as I describe in my comments above, such that the initial focus of the paper is more on the diversity of PMRs/mate guarding (chemical or physical) in flies, and less about the shared cosmopolitan ecology between mel and hydei, which is not discussed much in the discussion section.

Response: Thank you. We rearranged much of the manuscript and changed the emphasis throughout. We hope that the changes improve the manuscript to your satisfaction.

-The methods section (lines 550-551) notes that the same (apparently single) male was used for female remating in the fertility assays described in Fig. 1. Since this assay found no significant difference between females mated singly vs. multiply, I am wondering whether a potential explanation could be sperm exhaustion of the single male. Would the results be different if the females remated with fresh males instead? Or is there literature on D. hydei sperm exhaustion that could negate this issue? I would recommend the authors address this experimental design choice explicitly in the relevant part of the results section (lines 125-128).

Response: We considered this too, but Markow has previously shown that *D. hydei* males are capable of maintaining constant sperm transfer for at least 6 rematings. We added this precision in the Results section.

-Fig. S2: What is a "non-parametric t-test"? I think t-tests are parametric by definition, so please state the name of the actual non-parametric test used (e.g., Mann-Whitney U?).

Response: Sorry about that. Miscommunication led to the description of a Mann-Whitney as the "non-parametric version" of the t-test.
The test used here is a Mann-Whitney test. We corrected this.

Reviewer #3 (Remarks to the Author):

This manuscript is a valuable study that fills a gap in the literature regarding the post-mating responses of Drosophila hydei. This is a species which lack in Sex Peptide, a protein that induces the most known post-mating responses in many of the Sophophora group species. There's a lot of interesting data in this paper and it's well worth publishing. The data will be very useful for researchers in the field going forward and it is a rich dataset. Overall, the paper is pretty well written and easy to read, but there are a number of ways in which it could potentially be improved.

Response: Thank you for your kind review.

General comments:

• The abstract seems to focus on a rather small subsection if the results. It's a shame more of the nice findings aren't articulated here (or hinted at in the title); it risks that the paper will get often overlooked in literature searches when it actually contains lots of things that would interest a wide audience.

Response: Given the broad categories of experiments, we found it difficult to place everything in to the 250 word limit, while keeping its readability. I hope that we succeeded.

• The paper could be much more succinct. The results section is extremely wordy and encompasses a lot of discussion. As such when we get to the discussion, there is a lot of repetition making an overall length of the paper much longer than it really needs to be. A combined results and discussion section would be better if the journal style allows it.

Response: We did this. Thank you for the suggestion.

• Some of the thinking around the actions of the ejaculate in D. melanogaster – at least from a neurogenetics perspective – is that the default female state is the mated state; virgins suppress egg laying and refractoriness, and SP releases that suppression. So one potential viewpoint would be that D. hydei partially lacks that virgin suppression state. This may be worth some discussion.

Response: True. Having worked on the iab-4/8 miRNA, we were quite familiar with this idea. We did not include mention of this in the previous version of the manuscript for simplicity, but we now added a mention of it in the final parts of the discussion.

• Overall, there's a strong effort emphasis on the differences between species, but there are also quite a lot of similarity between the species. The PMR similarities might be regarded as quite surprising, given the very different mating system and sperm length. The paper would, IMO, be better balanced if it recognised both the similarities as well as the differences.

Response: I hope the new version is better in this respect, but our feeling is that the flies do respond quite differently to mating. However, it is a bit more nuanced than just that they

have different outputs. Basically, we think the flies are quite similar in overall biology, but that certain evolutionary changes, such as having giant sperm, fed into a similar "machinery" can lead to different outcomes. We hope this is satisfactory.

• *The statement that "D. hydei do not increase egg production" seems a little misleading. Very clearly mating does lead to an increase in egg production, it's just that it's over a short period of time, and followed by a decrease relative to virgins. In some ways that it's not so dissimilar to D. melanogaster, where at least in some strains virgin females will begin laying eggs at quite a high rate as they get older (there is genetic variability in this: some populations retaining eggs as virgins more strongly than others). At some point virgin egg laying overtakes that of mated females (e.g. day 16 in one of the experiments in this paper https://doi.org/10.1016/0022-1910(86)90140-X ; there are lots of other 20th century papers that look at the same thing). So, it is certainly on a very different time scale in the different species – but it could be argued that it's not entirely distinct phenomenon.*
• *The fact that multiple mating does not further increase egg laying (Fig 1C) is also arguably consistent with, not contradictory to, D. melanogaster.*
• *"….consistent with the idea that mating does not trigger an increase in oogenesis but instead triggers a short-term egg laying response to release stored eggs". Don't D. melanogaster also do this as part of the PMR? The ovulin response? So again, in some respects it seems that the strategy is not so different between the species.*

Response to three points above: I do not know if this is just a question of point of view, focusing on the differences rather than similarities. For our part, we feel that the responses are quite different at least in terms of levels and would like the study to highlight the changes. However, we have tried to tone this down a bit and point out some similarities also. We hope this version will be more equitable in this respect.

• *Sample sizes seem to be given for some experiments but not others – make Ns clear for all the experiments*

Response: We corrected this.

• *Some rather sloppy language and grammar throughout (see minor comments). In particular when naming Drosophila melanogaster and Drosophila hydei, sometimes it is written as D. melanogaster/hydei and sometimes only like melanogaster/hydei. Please, write it as Drosophila melanogaster or Drosophila hydei the first time and D. melanogaster or D. hydei on the following ones.*

Response: Sorry about this point. We tried to correct all instances of this.

• *Why is sample size of virgins 3x more than mated in Fig 1b? Could you please clarify this difference in the main text?*

Response: We actually had females that were virgin but exposed to males, and females that were never exposed to males. Because that data ended up not being statistically different, we pooled them and forgot to specify that we did this. Thank you for pointing this out. We

now explain this in the text and provide an accompanying supplementary figure that shows the two virgin results.

• *Figure 2B. Is a t-test the best way of analysing P1 (paternity sired by the first male)?*

Response: We agree that the t-test was probably not the best test for this data as it was comparing means with data that was likely not parametric. We redid the statistics using a Kolmogorov-Smirnov test as a non-parametric comparison of the distributions and confirm a significant change in the first male ratio. We would have liked to use other tests, like a Wilcoxon-ranked sum test as we commonly use for D. melanogaster, however we lacked an appropriate number of samples to run this test. Despite the fact that *D. hydei* females will remate if left alone in mating pairs, this is less frequent than one might think. In our lab, some flies mate many times, but most mate once in a one to two hour period. Also, we found that mating was highly circadian, with flies mating well only for an hour or so after lights-on. The tube manipulation and highly circadian nature of mating in *D. hydei* made remating very infrequent within the time frame of the experiment. Interestingly, with *D. melanogaster*, the sperm competition assay is generally performed by mating flies on subsequent days. With *D. hydei*, we could not do this, as the males deposit much fewer sperm and females lay most of their eggs on the first day.

• *In the sperm competition experiment (see discussion Line 438-443) it is discussed that sperm from starved males is less competitive than that of unstarved ones, based solely on the progeny sired by the female. If sperm count has not been possible to measure it is difficult to disentangle if the response was mediated by postcopulatory competition or female choosiness. Please, further discuss this.*

Response: This is what we were trying to argue in the text. Perhaps we were not clear. We have rewritten the text substantially. In essence, we believe that the female is probably not deciding to keep or discard sperm based on the look of the male, but maybe instead using SFPs as a proxy.

Minor:

Response to minor points: We have substantially changed the manuscript making listing the individual changes pointless. However, when we modified the document, we took into account the points made and believe that we corrected each issue. Thank you very much for your time and effort.

• *gene names should be italicized* –

• *final sentence of the abstract is rather vague "These changes impacting characteristics like lifespan and sperm selection demonstrate the variety of choices that species can make and be successful in a given environment". Not sure what point this is supposed to make.* –

• *L33-34: this phrasing sounds rather group-selectionist: selection acts on individuals > groups > species.* –

• *L39. This is first mention of seminal fluid proteins in main text, so introduce the acronym SFPs here (instead of L39)* –

• *L39 onwards: use "D." instead of Drosophila when talking about species.* –

• L43. Here you're talking about the SP gene: make this clear and italicise SP
• L45 onwards: make sure "D." comes in front of melanogaster or hydei –
• L52 "debatably"? I think one would have to be quite contrarian to argue that female refractoriness was not advantageous to the previous male –
• L71 "Perhaps due to this selection for long sperm, a second significant difference from melanogaster is the loss of the SP gene". I don't follow the logic of how selection on sperm length should lead to the loss of SP –
• L79 D. hydei
• L92 "yet equally successful". What on is that supposed to mean? How do you rate the success of two different species? –
• L105. Specify if the second female you are comparing them to is virgin or not, as it might be confusing for the reader. –
• L201. Fewer, not less.
• L214 "IV – The absence of SP has no direct consequences on the Male Accessory Gland shape or structure". This seems somewhat of an overinterpretation. Given that no direct manipulation of SP has been performed, I would change this title, or at least talk circumspection about associations. -
• L219 D.
• L258. True but SP is not made in the SCs. –
• L303 D.
• L340, In D. melanogaster, SP
• L343-345. This sentence is incomplete and doesn't make sense. • L379 D.
• Line 380. Drosophilids -> drosophilids
• L383 SP
• Line 384. "Drosophila" should be in italics
• Line 388. "Drosophila" should be in italics
• L389 D.
• Line 440. "melanogaster" should go in italics
• Line 451. "melanogaster" should be in italics
• L484 Whys suddenly Secondary Cells and not SCs?
• L492/3 sperm not sperms
• L506. "The differences of the PMR observed in D. hydei compared to what is known in D. melanogaster suggests that many reproductive strategies may be equally valid in the wild". Seems a rather meaningless statement. What does "valid" mean here?

Reviewer #4 (Remarks to the Author):

I co-reviewed this manuscript with one of the reviewers who provided the listed reports. This is part of the Communications Biology initiative to facilitate training in peer review and to provide appropriate recognition for Early Career Researchers who co-review manuscripts.
Response: Thank you for reading and reviewing our manuscript.

Dear Editors of Communications Biology

Please find uploaded the latest version of our manuscript entitled, “Characterization of the Post-Mating Responses of *Drosophila hydei*, a species that lacks Sex-Peptide”. We have attempted to respond to each of the reviewer remarks in turn and have noted our editions below. Finally, we would like to thanks each of the reviewers and the members of the Communications Biology team for helping us to improve our manuscrit.

REVIEWERS' COMMENTS WITH AUTHOR REPONSES:

Reviewer #2 (Remarks to the Author):

I appreciate the authors' careful attention to all of the reviewer comments. My main suggestion was to reconsider how the study was framed, and I think the new version is nicely improved in this area. I have just a few small suggestions, but feel the manuscript is now in good shape for publication. (I do not feel like I need to see the manuscript again.) Thanks to the authors for this interesting study, which I continue to feel will be an important one for the field.

Line 55-59: I really like the framing of the key questions here – thanks for this good revision! I also like the description (lines 73-91) of the interesting, relevant facets of D. hydei reproductive biology.

Line 287 and Fig. 4: The text says that AG lobe length was “no more than 1000 um” for the Sophophora group, but in Fig. 4B, it seems that a good number of the data points in the yellow-shaded region have lengths between 1000-2000 um. Please double check.

Indeed, thank you for spotting this error. The limit usually observed is around 1,600 µm. We corrected this oversight and added a precision about the general range.

Line 511-513: In addition to changes in the location and copy number of the SP gene in the melanogaster group, I believe a finding of the Tsuda et al. paper (ref 16) was that the SP receptor (SPR) also became much more highly expressed in the female reproductive tract within the melanogaster group. This might also be a contributing factor to why SP plays a more central role in mel-group species. Perhaps this is also worth a brief mention at this point in the discussion?

That’s an interesting point, we added a sentence about this.

Small editing suggestions:
Line 41: consider changing “have been found” to “have been identified”, since “found” was used previously in the sentence

Line 44: check for consistency across the manuscript in how male accessory gland is abbreviated (mAG vs. MAG).
Line 131-133: Consider inverting the order to better match the experimental result: this means D. hydei females will usually have a stock of eggs to lay upon mating (consistent with the observed uptick in laying in the first 24 hrs), but might occasionally have only a few available
Line 171-2: consider rewording “males or the act of constant mating” to “constant exposure to males or more frequent mating”
Line 205: “sperm seems” --> “sperm seem”
Line 358-9: check sentence fragment at the end

Reviewer #4 (Remarks to the Author):

I believe the authors have enhance the manuscript by implementing the suggestions made by the reviewers. I, therefore, think this paper is worth of publishing. Please see some minor comment bellow.

-The abstract has been rewritten showing a wider understanding of the results found in this study, which will potentially catch the attention of more readers.
-The length of the paper is still quite long, however we believe that the amount of data gathered in the study is a lot and needs to be explained. I thank the authors for joining the results and discussion sections making it less repetitive. Unless the editorial have any concerns with the length, we think is has been improved.
-There is more balance now throughout the ms between differences and similarities in both species.
-Language is much better now.
-Some of the general comments and minor comments have been addressed.

-Previous comment: “Some of the thinking around the actions of the ejaculate in D. melanogaster – at least from a neurogenetics perspective – is that the default female state is the mated state; virgins suppress egg laying and refractoriness, and SP releases that suppression. So one potential viewpoint would be that D. hydei partially lacks that virgin suppression state. This may be worth some discussion.”

I can’t see this has been explained somehow and it could be a very interesting point to further develop.

This issue was discussed in a couple of sentences around line 485.

-Previous comment: “The statement that “D. hydei do not increase egg production” seems a

little misleading. Very clearly mating does lead to an increase in egg production, it's just that it's over a short period of time, and followed by a decrease relative to virgins. In some ways that it's not so dissimilar to D. melanogaster, where at least in some strains virgin females will begin laying eggs at quite a high rate as they get older (there is genetic variability in this: some populations retaining eggs as virgins more strongly than others). At some point virgin egg laying overtakes that of mated females (e.g. day 16 in one of the experiments in this paper https://doi.org/10.1016/0022-1910(86)90140-X ; there are lots of other 20th century papers that look at the same thing). So, it is certainly on a very different time scale in the different species – but it could be argued that it's not entirely distinct phenomenon."

We added a bit more nuance in our description of the lack of increased egg production. It is true that in specific cases, and specific strains, virgin females may be observed laying more than, or as much as, mated females. We believe however that in our experimental design, the daily number of eggs observed in the weeks following a single mating event implies that females do not increase the amount of eggs that they produce.

Alternatively, what we see might be: a very short induction of egg production, followed by a strong inhibition of egg production. Such a dynamic, however, would require the evolution of two distinct mechanisms, one to increase egg production and one to decrease it, while our hypothesis only requires the mechanism to increase egg production, that is already known to exist in all other species studied, not to evolve.

-The title of the first result "Mated females show a short-term egg laying burst with no increase in egg production" is still misleading, however later the text they argue that it is a short-term effect and there are similarities between D. hydei and D. melanogaster.

We believe that we answered this comment by answering the previous one.

-Previous comment: "Why is sample size of virgins 3x more than mated in Fig 1b? Could you please clarify this difference in the main text?"

I can't see this in the new ms

We apologize for this, a paragraph previously added was lost in the reframing of the paper. A new paragraph has been added (lines 125 to 129).

-Previous comment: "In the sperm competition experiment (see discussion Line 438-443) it is discussed that sperm from starved males is less competitive than that of unstarved ones, based solely on the progeny sired by the female. If sperm count has not been possible to measure it is difficult to disentangle if the response was mediated by postcopulatory competition or female choosiness. Please, further discuss this."

I don't think this has been done, I would be more circumspect about this assumption.

Because of starvation causes many changes in the body of the fly, we tried to design the experiment to only look at sperm competition. Perhaps this was unclear in our materials and methods, but all matings were visually witnessed and only data from flies producing the two types of progeny were analyzed. In this way, we could be sure that the two types of sperm were present in the female, thus removing the choosiness variable (to the extent possible). We have added a sentence to the materials and methods to clarify this further as well as specifying this in the text (line 225).

New minor comments:

• 54. Change 'Drosophilids' to 'drosophilids'

• Line 230-240. Some this sentence has been included in the title IV, I assumed by mistake. – I don't see what they mean here.

• Line 487-489. The title 'V-Outlook on the reproductive strategy of D. hydei' has different fonts
